# DNAAF19–RUVBL1/2 complex recruits multiple adaptors to promote dynein arm assembly

**DOI:** 10.1073/pnas.2612755123

**Published:** 2026-10-06

**Authors:** Mafalda de Almeida Gomes, Emma Wood, Thomas Burgoyne, Franziska Klose, Denisa Batrinu, Karsten Boldt, Glenn Carrington, Yehuda Halfon, Rachel George, G. Nasir Khan, Tina Beyer, Hannah M. Mitchison, Takashi Ochi

**Affiliations:** ^a^ Genetics and Genomic Medicine Department, University College London, Great Ormond Street Institute of Child Health, University College London, London WC1N 1EH, United Kingdom; ^b^ Astbury Centre for Structural Molecular Biology and School of Molecular and Cellular Biology, Faculty of Health and Life Sciences, University of Leeds, Leeds LS2 9JT, United Kingdom; ^c^ Institute of Ophthalmology, University College London, London EC1V 9EL, United Kingdom; ^d^ Department of Paediatric Respiratory Medicine, Department of Paediatric Respiratory Medicine, Royal Brompton Hospital, Guy’s and St Thomas’ NHS Foundation Trust, London SW3 6NP, United Kingdom; ^e^ Core Facility for Medical Proteomics, University of Tübingen, Tübingen 72076, Germany; ^f^ Institute for Ophthalmic Research, Center for Ophthalmology, University of Tübingen, Tübingen 72076, Germany

**Keywords:** motile cilia, dynein assembly, DNAAF, primary ciliary dyskinesia, cryo-EM

## Abstract

Cilia are microtubule-based organelles essential for sensing, motility, and fluid transport. Motile cilia rely on inner and outer dynein arms, large multiprotein motor complexes, for force generation and the characteristic bending motion required for ciliary beating. Before being incorporated into cilia, these dynein arms are preassembled in the cytoplasm by dynein axonemal assembly factors (DNAAFs). Defects in this assembly pathway often cause the inherited genetic condition primary ciliary dyskinesia (PCD), in which the loss of dynein arms impairs ciliary motility. The molecular mechanisms governing the precise and timely assembly of dynein arms by DNAAFs remain elusive. Here, we investigated the role of dynein axonemal assembly factor 19 (DNAAF19), a DNAAF frequently mutated in PCD patients with combined inner and outer dynein arm defects. Using biochemical, structural, and proteomic approaches, we find that DNAAF19 is a monomeric protein that interacts directly with the heterohexamer of RUVBL1 and RUVBL2, a multifunctional cochaperone complex involved in ciliary dynein arm assembly. The recurrent PCD-causing variant p.His154Pro partially destabilizes the interaction of DNAAF19 with RUVBL1-RUVBL2. Moreover, we identify Deleted in Primary Ciliary Dyskinesia forming a cocomplex with RUVBL1/2-DNAAF19 in vivo and in vitro. This indicates similarity to R2TP, the HSP90 cochaperone composed of RUVBL1/2-RPAP3-PIH1D1 that assembles different molecular machineries in cells, and to the RUVBL1/2–SPAG1–PIH1D2 complex associated with ciliary dynein arm assembly and PCD. Our results suggest that DNAAF19 acts as a DNAAF in concert with RUVBL1/2 and its interacting proteins, providing insights into the molecular coordination of dynein arm assembly and cell biology of PCD.

## Significance

We identify dynein axonemal assembly factor 19 (DNAAF19) as a member of the proteins required for the correct assembly of dynein arm motors in motile cilia. Defects in these proteins cause primary ciliary dyskinesia, where cilia are immotile due to impaired motor assembly. We show *DNAAF19* expression increases as airway cells develop motile cilia and that DNAAF19 forms a complex with RUVBL1/2, a multifunctional chaperone, and DPCD (deleted in primary ciliary dyskinesia), as observed in vitro by cryo-EM. We further show that a prevalent *DNAAF19* disease-causing variant (p.His154Pro) destabilizes, but does not abolish, its interaction with RUVBL1/2. We propose that DNAAF19 functions as part of a cochaperone complex with RUVBL1/2 and other partners, including DPCD, to facilitate dynein arm assembly.

Motile cilia and structurally related sperm flagella are large, membrane-bound hair-like organelles extending from the cell surface. With an internal microtubule-based axoneme skeleton and accessory structures comprising hundreds of different proteins, they collectively perform the essential functions of generating fluid flow and cellular locomotion. Motile cilia reside on specialized epithelia of the respiratory tract, brain ventricles, fallopian tubes and uterus, epididymis, and embryonic node. Their biological and medical significance is marked by the human genetic conditions primary ciliary dyskinesia (PCD) and multiple morphological abnormalities of the sperm flagella (MMAF), caused by variants in genes governing cilia and sperm flagella generation, structure, and function (1, 2). A major question in the field of motile cilia research is how cilia are assembled in a timely and correct manner (3–5).

The structure of the motile cilium is highly conserved across eukaryotes, allowing motility within liquid environments by a continuous, self-generated forward and backward whip-like motion, generating fluid flow that aids in cellular movement and substance transport (6). The key component necessary for ciliary beating is the dynein arm, a multiprotein complex consisting of >20 different proteins, with the AAA+ ATPase dynein motor protein as the primary element (7). The inner (IDA) and outer (ODA) dynein arms, which have distinct structures, are regularly aligned along the axonemal microtubules and work together to slide the microtubules relative to one another. This sliding action bends the axonemes of the cilia, producing their characteristic beating motion, with ODAs providing the power and IDAs providing the waveform of the ciliary beat (8).

Dynein arms are initially assembled in the cytoplasm and then transported to the axonemes through a process known as intraflagellar transport (IFT) (9). The proteins involved in this assembly process are called dynein arm assembly factors (DNAAFs), with around 20 different proteins identified to date (10). The crucial roles of DNAAFs in assembling dynein arms are underscored by multiple pathogenic variants reported in ciliopathy patients, particularly those with PCD, which is characterized by respiratory, fertility, and other disease features reflecting motile cilia deficiency (11). Variants in at least 14 DNAAF genes are considered to cause PCD (2). Electron microscopy images of the axonemes from PCD patients carrying variants in DNAAFs typically reveal the absence of one or both (inner/outer) dynein arm types, confirming the involvement of DNAAFs in the dynein assembly process (1).

Dynein arm preassembly occurs in the cytoplasm through the coordinated action of multiple chaperone and cochaperone complexes. The R2TP complex, composed of RUVBL1, RUVBL2, RPAP3, and PIH1D1, works together with prefoldin-like subunits to form the PAQosome (Particle for Arrangement of Quaternary structure), a specialized HSP90 (Heat Shock Protein 90) cochaperone complex that mediates the assembly and stabilization of large macromolecular complexes, including axonemal dyneins (12, 13). Several DNAAFs, such as DNAAF2, DNAAF4, and SPAG1, have been shown to interact with R2TP-PAQosome components, supporting a conserved chaperone-guided mechanism for dynein arm biogenesis (14–16). *DNAAF2*, *DNAAF4*, and *SPAG1* all encode cytoplasmic proteins (17, 18) and pathogenic variants in these genes cause PCD (17, 19, 20). Understanding how individual DNAAFs function within this PAQosome network is essential for defining the sequence of molecular events that drive dynein arm assembly. Recent work has further suggested that this chaperone-guided preassembly is spatially organized within specialized, liquid-like cytoplasmic compartments termed dynein axonemal particles (DynAPs), which concentrate DNAAFs, dynein subunits, and associated chaperone machinery prior to intraflagellar transport (21).

In humans, the differentiation of airway epithelial cells into multiciliated cells can be modeled in vitro using air–liquid interface (ALI) culture. In this system, primary human nasal epithelial cells are expanded under submerged conditions and subsequently exposed to air by removing apical media, triggering multiciliogenesis that recapitulates in vivo cilia formation (22). ALI culture allows cells to be collected at different stages as they differentiate from basal cells into a fully ciliated epithelium, enabling the measurement of changes in expression of DNAAFs and dynein subunits starting from when the motile airway cilia begin to form.

Dynein axonemal assembly factor 19 [DNAAF19; until recently known as Coiled-Coil Domain-Containing protein 103 (CCDC103)] is one of the DNAAFs initially identified as mutated in individuals with PCD and airway ciliary axonemes exhibiting a lack of both inner and outer dynein arms (23). The most frequent pathogenic *DNAAF19* variant is a founder allele that predominates in the UK and other PCD cohorts, causing a single amino acid change or “missense” variant (c.461A>C; p.His154Pro or H154P) (24). The H154P variant confers a partial loss-of-function effect associated with partial-to-minimal dynein arm loss phenotype, whereas loss-of-function null allele variants confer a more clear-cut loss of both dynein arms (23, 25). Past biochemical studies suggested that DNAAF19 could be an axonemal docking protein for outer dynein arms (26). However, recent cryoelectron microscopy (cryo-EM) structures of ciliary axonemes have not revealed the presence of DNAAF19, leaving its role in dynein arm assembly unclear (27).

Here, we show that DNAAF19 is a DNAAF that interacts with the heterohexamer of RUVBL1 and RUVBL2 (RUVBL1/2), an AAA+ ATPase playing ubiquitous roles in cells (13). Using the soluble form of DNAAF19, we show that DNAAF19 is a monomeric protein that does not directly interact with microtubules. In human airway ALI cell cultures, *DNAAF19* is up-regulated during multiciliogenesis and expresses a cytosolic protein product, similarly to *SPAG1*. Using SF-TAP affinity purification followed by mass spectrometry, we find that DNAAF19 interacts with R2TP/PAQosome components in vivo, including RUVBL1/2. We show that this resembles how RPAP3 interacts with the hexamer in vitro using cryo-EM, and that Deleted in Primary Ciliary Dyskinesia (DPCD) can form a cocomplex with the DNAAF19–RUVBL1/2 complex. These results suggest that DNAAF19 is a DNAAF-type HSP90 cochaperone participating in dynein arm assembly through interaction with RUVBL1/2.

## Results

### DNAAF19 Is a Monomeric Protein that Localizes to the Cytoplasmic Subapical Region in Airway Epithelial Cells.

**Fig. 1.**
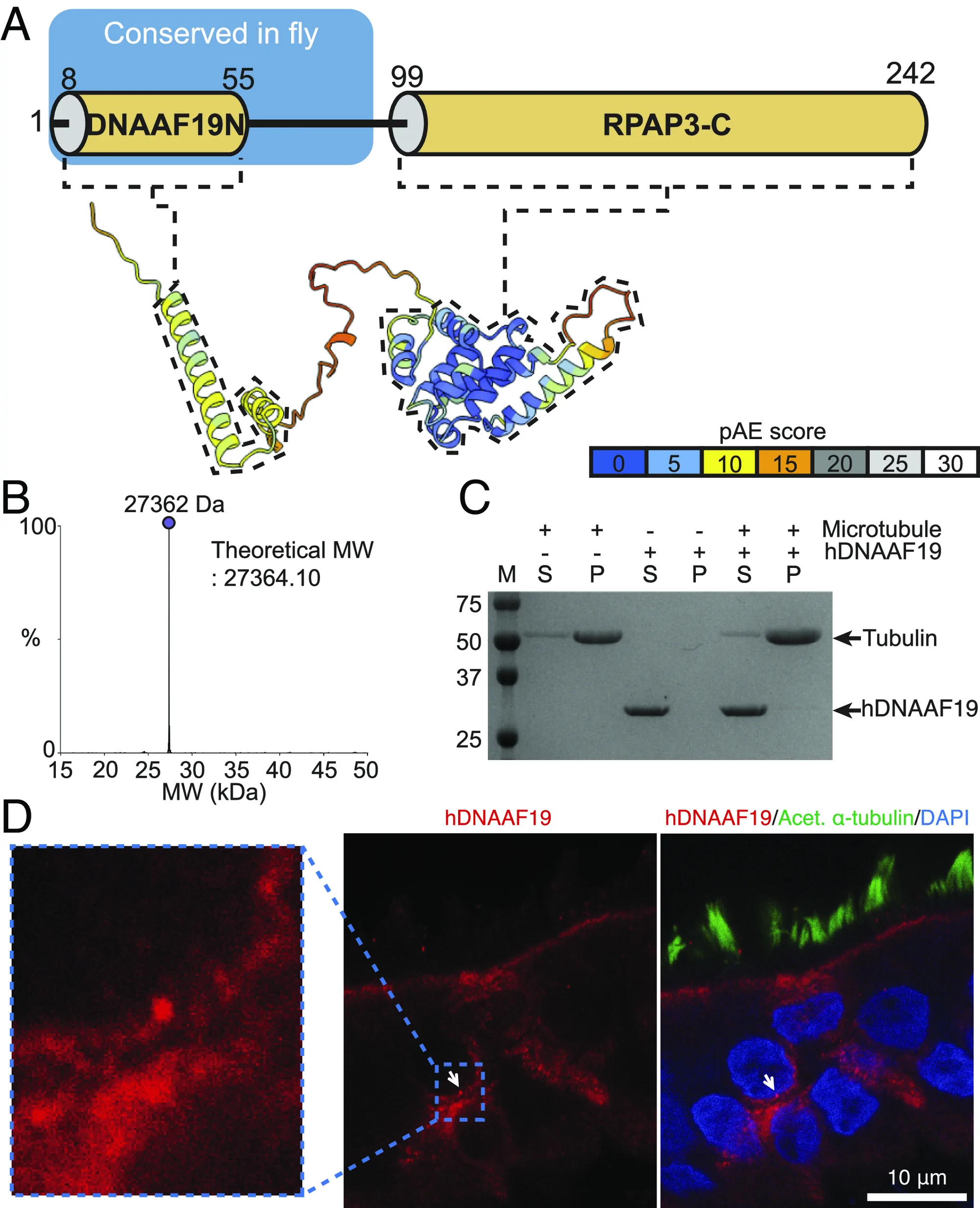
hDNAAF19 is a monomeric protein that does not interact with microtubules. (*A*) AlphaFold model of hDNAAF19. The chain was colored based on the pAE score of residues. The DNAAF19N domain conserved in *Drosophila* is highlighted. (*B*) Native mass spectrometry of hDNAAF19. A single peak at 27 kDa was observed from purified hDNAAF19. (*C*) Microtubule-pelleting assay for hDNAAF19. An SDS-PAGE gel shows the microtubule-pelleting assay result of hDNAAF19. M, S, P, +, and − indicate the molecular ladder, supernatant, pellet, and presence and absence, respectively. The numbers on the left are molecular weights (kDa). (*D*) DNAAF19 localizes to punctate cytoplasmic structures with apical enrichment in human airway epithelial cells. Immunofluorescence staining of cryosectioned human airway epithelial cells shows DNAAF19 (red) exhibiting a punctate cytoplasmic localization, including perinuclear foci (white arrow), with enrichment in the apical cytoplasm immediately beneath the ciliary layer marked by acetylated α-tubulin (green). Nuclei are counterstained with DAPI (blue). The *Left* panel shows the DNAAF19 channel alone, with an enlarged view outlined in dashed blue lines in the *Inset* to the *Far Left*.

The primary structure of human DNAAF19 (hDNAAF19) was analyzed computationally to gain structural insight into its function. AlphaFold3 (28) modeling showed that it has an N-terminal helical domain (DNAAF19N) followed by a C-terminal RPAP3-C domain (Fig. 1*A*). This is homologous to the RPAP3-C domain found in the C terminus of RPAP3 and SPAG1, that is known to interact with RUVBL1/2 heterohexamer (29). This model and its analysis in Foldseek (30) indicated that DNAAF19N is unique to DNAAF19 only. Although DNAAF19 shares the RPAP3-C domain with RPAP3 and SPAG1, it is distinct in lacking the tetratricopeptide repeat (TPR) domains found in RPAP3, SPAG1, and various cochaperones, that allow them to bind to the molecular chaperone HSP90 to regulate its activity (29). Instead, DNAAF19 has the unique N-terminal DNAAF19N region, which notably is its only conserved domain in the DNAAF19 *Drosophila* ortholog (Fig. 1*A*). Thus, DNAAF19N likely has the essential, evolutionarily conserved function of DNAAF19, while its RPAP3-C domain is critical but may also have a redundant role, possibly in conjunction with RPAP3 or SPAG1.

Previously, hDNAAF19 and its *Chlamydomonas* ortholog expressed in bacteria were purified from inclusion bodies, and their biochemical analyses suggested that they were multimers that interact with microtubules (23, 26). Although we tried modeling hDNAAF19 homodimer using AlphaFold3, we could not obtain reliable homodimer models (ipTM = 0.238). The previous observation of hDNAAF19 multimers could be due to misfolding of the refolded recombinant hDNAAF19. We therefore established a protocol to purify soluble recombinant hDNAAF19 using the lipoyl tag, which is the lipoyl domain of the E2p protein in *Bacillus stearothermophilus* (31), and revisited those previous results using the soluble form of recombinant hDNAAF19. As expected from the AlphaFold model (Fig. 1*A*), circular dichroism analysis of the purified hDNAAF19 protein showed an α-helical profile and a melting temperature of 58.4 °C (*SI Appendix*, Fig. S1*A*). Despite our efforts to purify the H154P mutant of hDNAAF19 (hDNAAF19^H154P^) using the same approach as the wild-type, we were unable to do so because the mutant precipitated mostly after removing the lipoyl-tag. This suggests that the H154P variant destabilizes hDNAAF19 in vitro. We observed the hDNAAF19 monomer only using native mass spectrometry (Fig. 1*B* and *SI Appendix*, Fig. S1*B*). Moreover, microtubule-pelleting assays of hDNAAF19 showed no interaction between it and microtubules (Fig. 1*C*). Therefore, contrary to the previous observation, our results using soluble hDNAAF19 suggest that hDNAAF19 is a monomeric protein that does not bind microtubules. This suggests that hDNAAF19 is unlikely to work as an outer dynein arm docking protein.

We investigated hDNAAF19 cellular localization in cryosections of differentiated airway epithelium grown in ALI culture from basal cells of a healthy human subject. Immunofluorescence staining with a hDNAAF19-specific antibody (*SI Appendix*, Fig. S2) revealed a cytosolic, punctate localization with perinuclear puncta and subapical enrichment beneath the multicilia (Fig. 1*D*). This punctate cytoplasmic distribution echoes that of other DNAAFs including DNAAF2, DNAAF5, and SPAG1 (16, 18), suggesting that DNAAF19 also resides in the cytoplasmic compartment where dynein arm components are assembled before being transported into the cilium. Such localization is consistent with a role as a DNAAF, in line with SPAG1 and other DNAAFs that act in proximity to the basal bodies to facilitate dynein preassembly and delivery to the axoneme (21). Similar perinuclear puncta were observed in both immature and mature ciliated airway epithelial cells (*SI Appendix*, Fig. S3), indicating that this localization pattern is established during ciliogenesis and maintained following ciliary maturation.

### Dnaaf19 Interactome Reveals Associations with R2TP/PAQosome Machinery and Dynein Assembly Components.

To understand the role of DNAAF19 in dynein arm assembly, we characterized the protein–protein interaction network of mouse Dnaaf19 using SF-TAP affinity purification followed by mass spectrometry. Capturing the tissues where Dnaaf19 is expressed most strongly, pulldowns were performed using wild-type mouse lung and testis lysates incubated with SF-TAP-tagged wild-type Dnaaf19 (WT) or Dnaaf19^H154P^ variant (MUT) baits, with a nonciliary Raf1 (Rapidly Accelerated Fibrosarcoma 1) pulldown serving as a negative control. Principal component analysis revealed a clear separation between replicate experiments on two experimental days, reflecting variability in the background protein composition of tissue lysates obtained from different mice used for each experiment (*SI Appendix*, Fig. S4). To minimize the impact of this variability, subsequent analyses focused on proteins that were consistently and significantly enriched or altered across both experimental replicates.

**Fig. 2.**
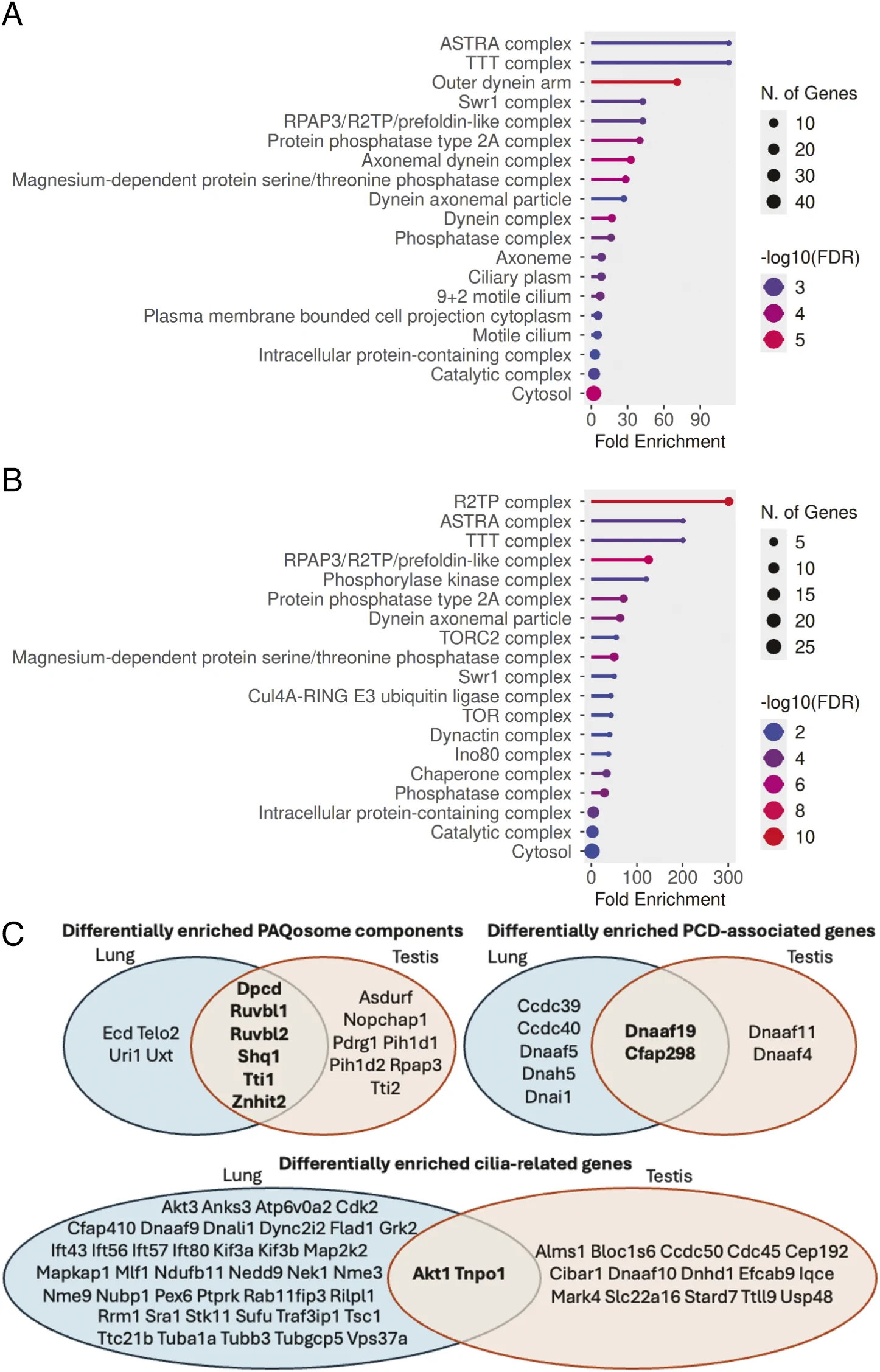
Dnaaf19 association with R2TP/PAQosome machinery, dynein assembly, and other cilia-related components. (*A*) GO enrichment of DNAAF19-interacting proteins in the mouse lung. Gene Ontology Cellular Component enrichment analysis (ShinyGO) identified proteins significantly altered in both WT vs. control and WT vs. MUT comparisons. (*B*) GO enrichment of Dnaaf19-interacting proteins in mouse testis. ShinyGO enrichment analysis identifies proteins significantly altered in both WT vs. control and WT vs. MUT comparisons. Bubble sizes indicate the number of genes per term; color represents –log_10_ (FDR). Terms related to dynein arm assembly and PAQosome/R2TP complexes were among the most significantly enriched. (*C*) Summary of tissue-specific comparison of Dnaaf19 interactors in lung vs. testis. Venn diagrams depict proteins significantly enriched in either WT vs. control, WT vs. MUT, or both comparisons. Proteins were categorized by three functional classes: PAQosome-related components; PCD/dynein arm assembly proteins, and other cilia-related proteins. Shared interactors represent elements of the Dnaaf19 interactome shared across both tissues, whereas nonoverlapping regions highlight tissue-specific cofactor utilization.

We identified the classes of mouse Dnaaf19-interacting proteins significantly enriched in pulldowns for both Dnaaf19 WT vs. control and WT vs. MUT comparisons using ShinyGo, the gene ontology (GO)-based cellular component enrichment analysis tool. This highlighted an overall enrichment of Ruvbl1/2-related cochaperone machinery as Dnaaf19-interacting proteins in both lung (Fig. 2*A*) and testis (Fig. 2*B*) datasets. Proteins classified by GO terms related to axonemal dynein arm complex components and PAQosome/R2TP complexes (“RPAP3/R2TP/prefoldin-like complex,” “ASTRA complex,” “TTT complex”) were among the most significantly enriched in both tissues.

**Fig. 3.**
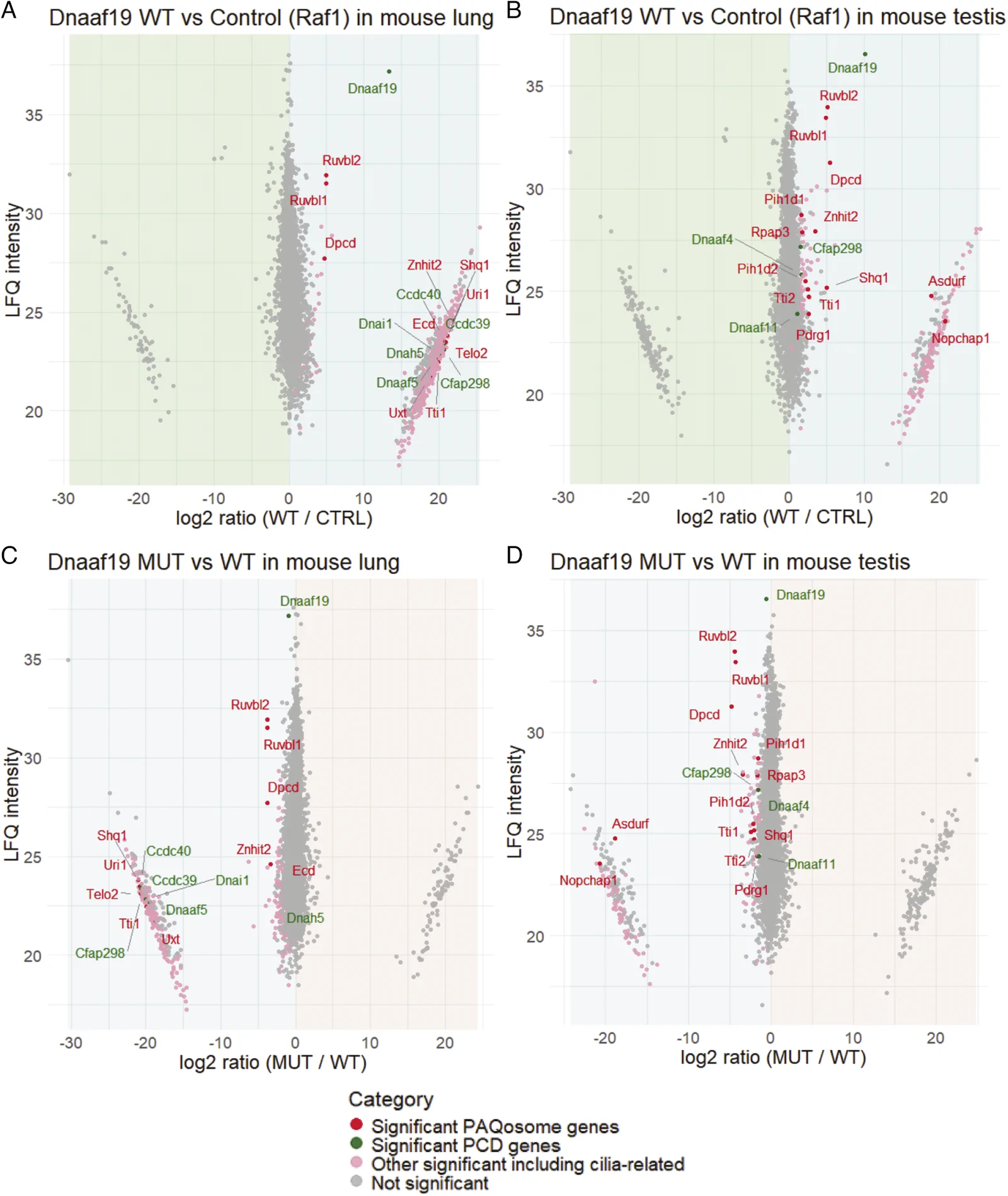
Dnaaf19 pulldowns highlight PAQosome and PCD-related protein partners. Differential interactome analysis of Dnaaf19 in mouse tissues: (*A*) WT Dnaaf19 vs. Raf1 control in lung; (*B*) WT Dnaaf19 vs. Raf1 control in testis; (*C*) H154P mutant (MUT) vs. WT in lung; and (*D*) H154P mutant vs. WT in testis. Each point represents a quantified protein based on label-free quantification (LFQ) intensity.

In WT vs. control pulldowns from both tissues, we observed strong enrichment of multiple R2TP/PAQosome-associated factors. This included Ruvbl1/2, Dpcd, and several R2TP-related assemblies involved in multiple “housekeeping” pathways including spliceosome (e.g., Znhit2), snoRNPs (e.g., Shq1), and PIKK cochaperone complexes (e.g., Tti1) (Figs. 2*C* and 3 *A* and *B*). Dpcd was prominent among Dnaaf19 interactors and has also been linked to ciliogenesis of primary (nonmotile) cilia, reported as an adaptor binding to R2TP regulating Akt kinase (32). Notably, Hsp90 (Hsp90aa1/Hsp90ab1) was not significantly enriched in either tissue, consistent with DNAAF19 lacking the TPR motifs required for direct Hsp90 binding (Fig. 1*A*). Thus, a subset of recovered PAQosome-associated proteins may reflect indirect copurification via Dnaaf19-associated Ruvbl-containing complexes, rather than a direct Dnaaf19–Hsp90 recruitment.

Beyond R2TP/PAQosome components, WT Dnaaf19 pulldowns revealed robust enrichment of a broad set of cilia-related proteins, including several whose dysfunction causes PCD, spanning multiple functional categories. These included dynein assembly factors (e.g., Dnaaf5, Dnaaf9, Dnaaf10, Dnaaf11, Cfap298), axonemal dynein components (e.g., Dnah5, Dnai1), intraflagellar transport proteins (e.g., Ift43, Ift56, Ift57, Ift80), centrosomal and basal body-associated proteins (e.g., Cep192), and additional cilia-related or ciliogenesis-associated factors (Figs. 2*C* and 3 *A* and *B* and *SI Appendix*, Fig. S5 and Tables S1 and S2). Many of these proteins were shared between lung and testis WT interactomes, indicating a conserved Dnaaf19-associated ciliary interaction network across tissues, while others showed tissue-specific enrichment. For example, fewer IFT components being enriched in testis pulldowns could reflect the IFT requirement in flagellum assembly but not mature sperm maintenance (33).

The cilia-related proteins identified in the pulldowns divided into cilia-localized components (basal body and/or axonemal, fewer membrane-associated), or cytosolic and ciliary base-localized proteins (*SI Appendix*, Tables S1 and S2). Axonemal proteins were mainly IFT, microtubule-associated, and dynein arm–related; the basal body proteins linked to structural, ciliogenesis, signaling, and trafficking functions; the cytosolic/ciliary base proteins have roles in vesicular transport, ciliary import, motility, dynein assembly, and signaling.

Comparison of WT Dnaaf19 vs. MUT Dnaaf19^H154P^ pulldowns revealed a marked reduction or loss of these interactions in both tissues (Figs. 2*C* and 3 *C* and *D* and *SI Appendix*, Fig. S5 and Tables S1 and S2), indicating that the H154P variant disrupts the interaction of Dnaaf19 with dynein, IFT, and R2TP/PAQosome components and compromises complex stability across tissues.

While a core subset of disrupted interactors was common to both tissues, a notable tissue-specific feature was the enrichment of Rpap3 in testis pulldowns but not in lung pulldowns. Given that Dnaaf19 contains an RPAP3-C domain, this pattern could reflect differential utilization of RUVBL1/2 adaptors across tissues, with different Dnaaf19 complex adaptor usage in ciliated airway epithelium vs. spermatogenic tissue. Collectively, the interactome data suggested that DNAAF19 functions as a DNAAF in association with the RUVBL1/2 complex and its interacting partners.

### DNAAF19 Is Coexpressed with PCD-Related DNAAF/PAQosome Assembly Factors During Human Airway Multiciliogenesis.

**Fig. 4.**
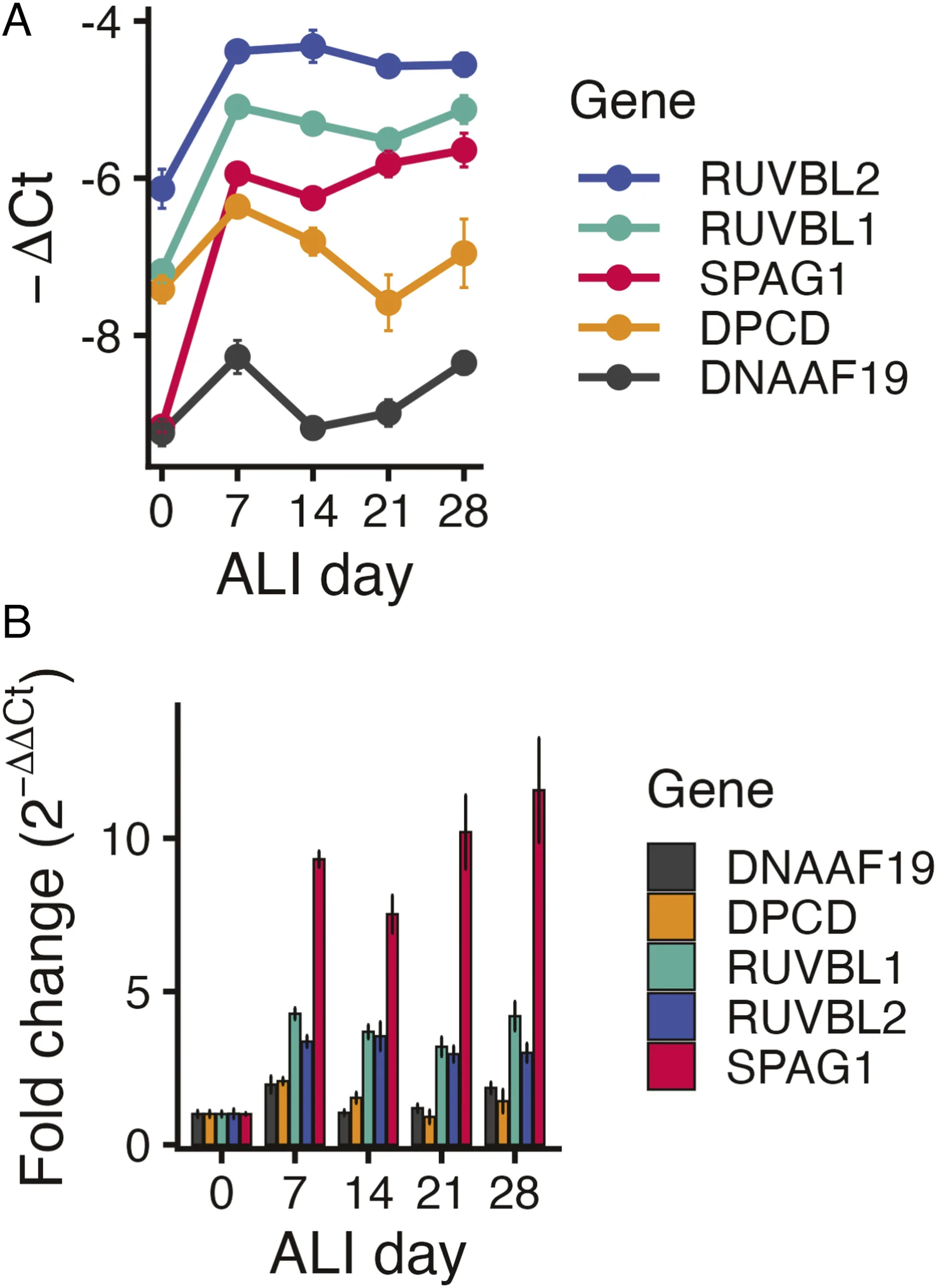
Baseline abundance and temporal induction of PAQosome and dynein assembly factors during ALI differentiation in airway epithelial cells. (*A*) Gene expression trajectories plotted as –ΔCt across ALI timepoints, highlighting coordinated transitions between basal cells (day 0) and early ciliogenesis (day 7). (*B*) Fold change in expression (2^–ΔΔCt^) relative to ALI day 0 for each gene across the differentiation time course. Bars represent mean ± SD from three biological replicates per timepoint.

To further explore this relationship in a relevant human airway differentiation model, we selected a focused subset of candidates for transcript analysis: RUVBL1 and RUVBL2 (core components of the most consistently enriched DNAAF19-associated complex), DPCD (prominent PAQosome-associated interactor linked to ciliogenesis), SPAG1 (known PCD-associated DNAAF and RPAP3-C-domain-containing protein comparator for DNAAF19 during dynein arm preassembly). These factors are relevant to the DNAAF19–RUVBL1/2 complex, motile cilia disease, or ciliogenesis. Control human airway epithelial cells were differentiated at ALI and sampled across defined timepoints. The expression of PAQosome genes notably enriched in Dnaaf19 pulldowns and reduced in the Dnaaf19^H154P^ pulldowns (*RUVBL1/2, DPCD)* was assessed, along with the PCD-associated DNAAF *SPAG1*, measuring both the relative abundance (–ΔCt, normalized to *GAPDH*) and the fold change relative to ALI day 0. At baseline, *RUVBL1* and *RUVBL2* were the most abundant transcripts (Fig. 4*A*), consistent with their roles in multiple cellular processes. *DPCD* also showed comparatively high baseline expression, indicating that these components associated with dynein assembly machinery but also having roles outside of motile ciliated cells are present in the airway basal cells prior to differentiation (32).

A coordinated shift in expression was observed between ALI day 0 and day 7 across all five assayed genes (Fig. 4*A*), coincident with initiation of the multiciliogenesis program. While *RUVBL1/2* and *DPCD* remained highly expressed throughout differentiation, *SPAG1* and *DNAAF19* both showed prominent induction during early ciliogenesis, consistent with SPAG1’s established role in dynein arm preassembly in differentiating multiciliated cells (16) and suggesting similar timing of *DNAAF19* induction.

Analysis of expression as fold change relative to day 0 further highlighted *SPAG1* as the most strongly induced factor across the differentiation time course (Fig. 4*B*). In contrast, *DNAAF19* remained low in absolute abundance, but like *SPAG1* this increased reproducibly during early differentiation and remained elevated thereafter. This pattern suggests that *DNAAF19* expression and function are temporally regulated during ciliogenesis akin to *SPAG1* and consistent with its proposed role in dynein arm assembly, but this may depend more on controlled complex assembly for DNAAF19 than on high transcript abundance. In contrast, *DPCD* expression more closely followed *RUVBL1/2* during multiciliogenesis.

### Asymmetric Stoichiometry of Copurified hDNAAF19–RUVBL1/2 Complex.

**Fig. 5.**
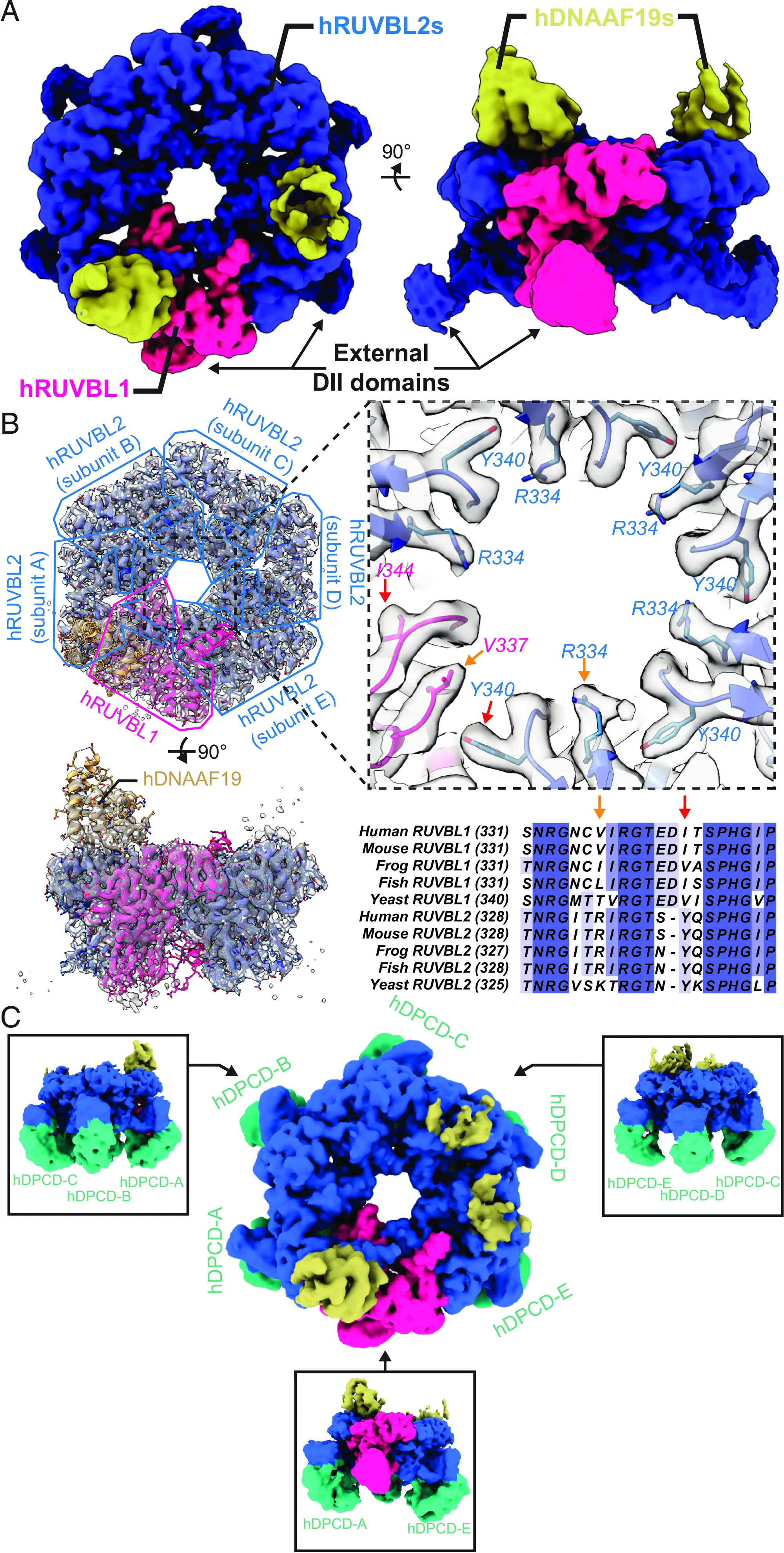
1:5 stoichiometry of the hRUVBL1/2 hexamer. (*A*) The cryo-EM map of the hDNAAF19–RUVBL1/2 complex. The map surface is colored by hRUVBL1 (magenta), hRUVBL2 (blue), and hDNAAF19 (yellow). (*B*) Structural models of hDNAAF19–RUVBL1/2 were fitted into the sharpened cryo-EM map. hRUVBL1, hRUVBL2, and hDNAAF19 are shown in a cartoon presentation and colored in magenta, blue, and light yellow. A magnified view within a dotted box highlights a region having distinct structural differences between hRUVBL1 and hRUVBL2. Below the dotted box, a sequence alignment of the highlighted region is presented. (*C*) Five copies of hDPCD bound to hRUVBL2. The composite cryo-EM map of the hDNAAF19–RUVBL1/2–DPCD complex was colored using the same color scheme as (*A*) in addition to hDPCD (aquamarine). The maps in black squares show side views from three different angles. hDPCD-A–E indicate hDPCD molecules that interact with the subunits A–E of hRUVBL2.

Our interactome studies suggest that DNAAF19 interacts with RUVBL1/2. We therefore copurified these three human proteins coexpressed in bacterial cells and determined the cryo-EM structure of the complex at an overall 3.5-Å resolution (Fig. 5*A* and *SI Appendix*, Fig. S6 *A*–*C* and Table S4). We observed one clear and one less well-resolved density of hDNAAF19 sitting on the top of the hexameric ring of hRUVBL1/2. The external RUVBL DII domains appear to be flexible (Fig. 5*A* and *SI Appendix*, Fig. S6*A*), as reported previously (34). We did not observe major 2D classes of hRUVBL1/2 dodecamers, which were observed in hRUVBL1/2 in complex with hRPAP3 or hSPAG1 (34–36). Interestingly, inspection of the cryo-EM map of the hDNAAF19–RUVBL1/2 complex suggests a 1:5 stoichiometry of hRUVBL1:RUVBL2 (Fig. 5*B*). This was apparent from the structural differences between hRUVBL1 and hRUVBL2, particularly in the sequence differences of residues 331 to 351 in hRUVBL1 and 328 to 347 in hRUVBL2 (Fig. 5*B*). Indeed, at a low map threshold level, we observed up to five copies of hDNAAF19, although the densities of hDNAAF19 molecules varied (*SI Appendix*, Fig. S6*D*). Focused 3D classification showed that hDNAAF19 is absent from hRUVBL1 but present in all five hRUVBL2 subunits with variable occupancy (*SI Appendix*, Fig. S7 *A* and *E*), preferentially associating with hRUVBL2 subunits interfacing with hRUVBL1 (notably subunits A and E). Furthermore, focused 3D classification of the DI, internal DII, and DIII domains (RUVBL core) of hRUVBL1 and the five hRUVBL2 subunits, following C6 symmetry expansion, revealed that one class exhibiting unique hRUVBL1 features accounts for ~17% of all particles (*SI Appendix*, Fig. S7*B*). This further supports the 1:5 stoichiometry of the hRUVBL1/2 hexamer.

Since the sequence identity between hRUVBL1 and hRUVBL2 is 43% (13), to clearly distinguish hRUVBL2 from hRUVBL1 in the cryo-EM map, we mixed the hDNAAF19–RUVBL1/2 complex with human DPCD (hDPCD). This was one of the top hits of our Dnaaf19 interactome analyses (Figs. 2 and 3) and is known to specifically interact with the DII domain of RUVBL2 (32, 37). We determined the cryo-EM structure of the hDNAAF19–RUVBL1/2–DPCD complex at 3.5-Å resolution (Fig. 5*C* and *SI Appendix*, Fig. S6 *A*–*C*). The EM map clearly showed five copies of hDPCD bound to hRUVBL2, and the density was absent in hRUVBL1 (Fig. 5*C*). Focused 3D classification on the density region corresponding to hDPCD and the external DII domain of hRUVBL2 revealed that hDPCD occupancy exceeds 92% across the five hRUVBL2 subunits, whereas it is only ~3% in hRUVBL1 (*SI Appendix*, Fig. S7 *C* and *E*). The map densities comprising this 3% lack well-defined structural features and are likely noise, indicating that hDPCD is absent from hRUVBL1. Consistently, hDNAAF19 is also absent from hRUVBL1 but present in the hRUVBL2 subunits (*SI Appendix*, Fig. S7 *C* and *E*). Furthermore, as observed for the hDNAAF19–RUVBL1/2 complex, focused 3D classification of the RUVBL cores of hRUVBL1 and hRUVBL2 following C6 symmetry expansion showed that ~17% of particles display the unique features of hRUVBL1, corresponding to its expected subunit stoichiometry (*SI Appendix*, Fig. S7*D*). Collectively, these results support that the predominant stoichiometry of hRUVBL1/2 in our hDNAAF19–RUVBL1/2 complex is primarily 1:5.

### DNAAF19 Interacts with RUVBL1/2 in a Similar Manner to RPAP3.

**Fig. 6.**
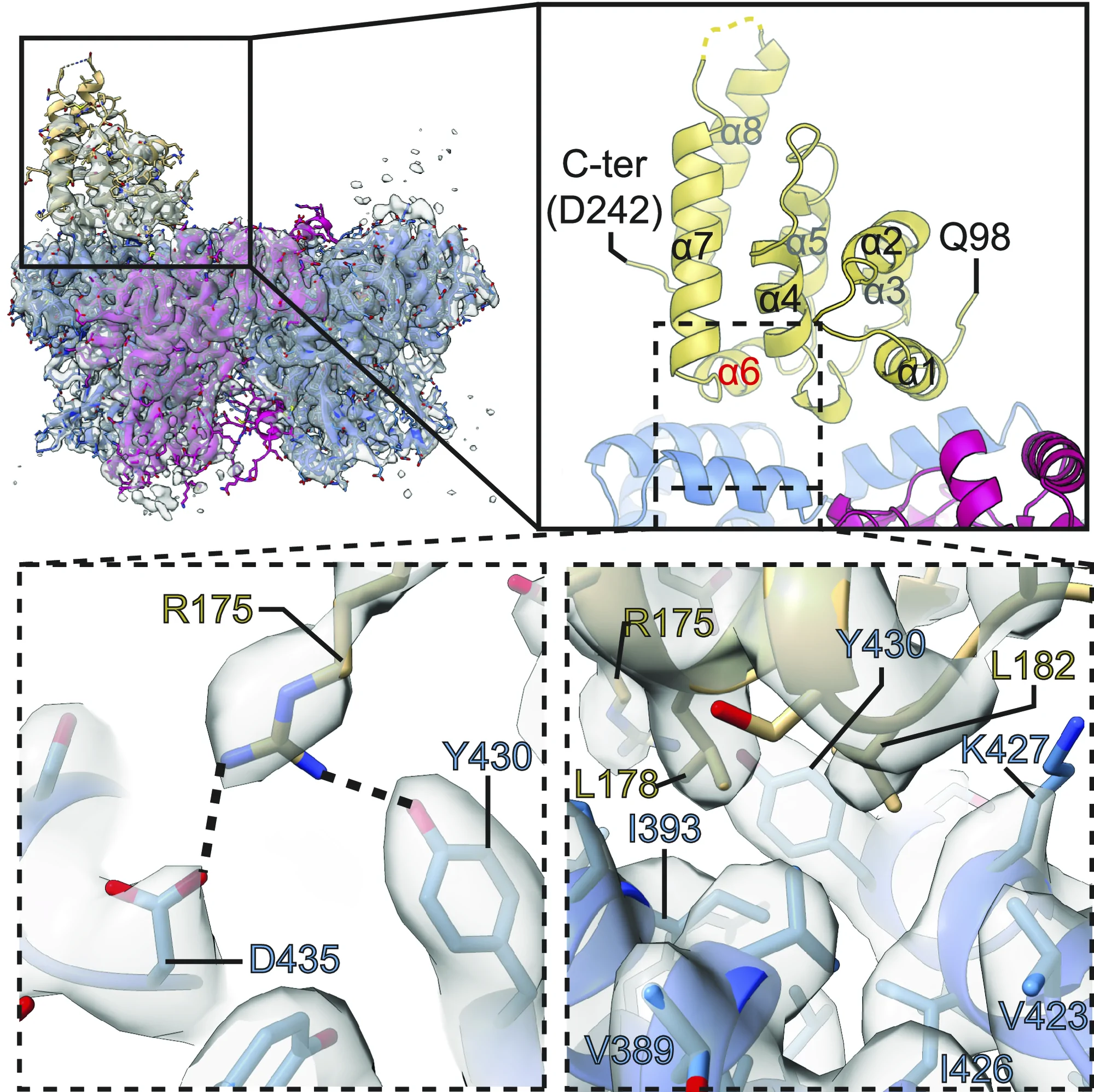
Interaction between hDNAAF19 and hRUVBL2. The direct interaction between hRUVBL2 and α6 of the RPAP3-C domain of hDNAAF19. The structural model and map of the hDNAAF19–RUVBL1/2 complex shown in Fig. 5*B* was magnified to show the details of the protein–protein interaction interface between hDNAAF19 and hRUVBL2 in solid and dotted line squares.

The cryo-EM structure of hDNAAF19–RUVBL1/2 revealed that the RPAP3-C of hDNAAF19 interacts with hRUVBL1/2 in a manner similar to hRPAP3, with the external DII domains adopting an open conformation, as observed in the hRPAP3-RUVBL1/2 complex (Fig. 5*A*) (34). The interaction between hDNAAF19 and hRUVBL1/2 is primarily mediated by the α6 helix of RPAP3-C, where residue R175 forms salt bridges with D435 and Y430 (Fig. 6). Meanwhile, hDNAAF19 L178 and L182 engage a hydrophobic pocket formed by V389, I393, V423, K427, and Y430 of hRUVBL2 (Fig. 6). These contacts create an interaction interface of ~700 Å^2^ between hDNAAF19 and hRUVBL1/2, approximately 250 and 400 Å^2^ smaller than that of hRPAP3-RUVBL1/2 and hSPAG1-RUVBL1/2. The comparison of hDNAAF19, hRPAP3 (34), and hSPAG1 (35) interacting with hRUVBL1/2 revealed that the difference in their interaction interfaces can be explained by the contact angle of α1: that of hSPAG1 is nearly parallel to the hRUVBL1/2 surface, whereas those of hRPAP3 and hDNAAF19 are ~15 and 30°, respectively (*SI Appendix*, Fig. S8). No density was observed for hDNAAF19 before residue Q98, suggesting that DNAAF19N is flexibly tethered to hRUVBL1/2. The same interaction between hDNAAF19 and hRUVBL2 was also observed in hDNAAF19–RUVBL1/2–DPCD (Fig. 5*C*), suggesting that DPCD does not interfere with the interaction between DNAAF19 and RUVBL2.

We further explored the effect of the PCD-causing H154P variant on DNAAF19’s ability to form a complex with RUVBL1/2, as hDNAAF19^H154P^ had precipitated after removing the lipoyl tag, while wild-type hDNAAF19 was stable. AlphaMissense (38) also predicted the variant as ambiguous (*SI Appendix*, Fig. S9*A*). The structure of hDNAAF19 shows that H154 is located at the end of α4 and that it stabilizes the RPAP3-C domain by interacting with P117 (*SI Appendix*, Fig. S9*B*). The H154P variant destabilizes the RPAP3-C domain by losing the interaction, but the variant itself is unlikely to be structurally disruptive, as H154 is located at the end of α4, which means the residue does not need to take phi-psi angles within the α-helix region strictly. We studied whether hDNAAF19^H154P^ could still interact with hRUVBL1/2 by expressing it in HEK293T cells. We found that transiently expressed hDNAAF19^H154P^ still interacts with endogenous hRUVBL2, even if somewhat more weakly (*SI Appendix*, Fig. S9*C*). These results suggest that the H154P variant of hDNAAF19 is a hypomorphic variant that destabilizes the RPAP3-C domain but still retains the interaction with hRUVBL2.

We also mapped other PCD-causing variants of hDNAAF19 onto its structure (*SI Appendix*, Fig. S9 *A* and *B*). Since variants introducing premature stop codons are unlikely to produce functional protein, we focused on the R35P and L183P missense variants. AlphaMissense predicted both variants to be “likely pathogenic.” The R35P substitution is predicted likely to disrupt an α-helix within the N-terminal domain of hDNAAF19 (*SI Appendix*, Fig. S9*B*), whereas the L183P variant likely distorts the RPAP3-C domain by altering the conformation of the linker between α6 and α7 and disrupting a hydrophobic interaction involving L180. These data suggest that the correct folding of both DNAAF19 and the RPAP3-C domain is important for hDNAAF19 function, and that destabilization of either domain can lead to PCD.

### DPCD Facilitates Nucleotide Exchange of RUVBL2.

**Fig. 7.**
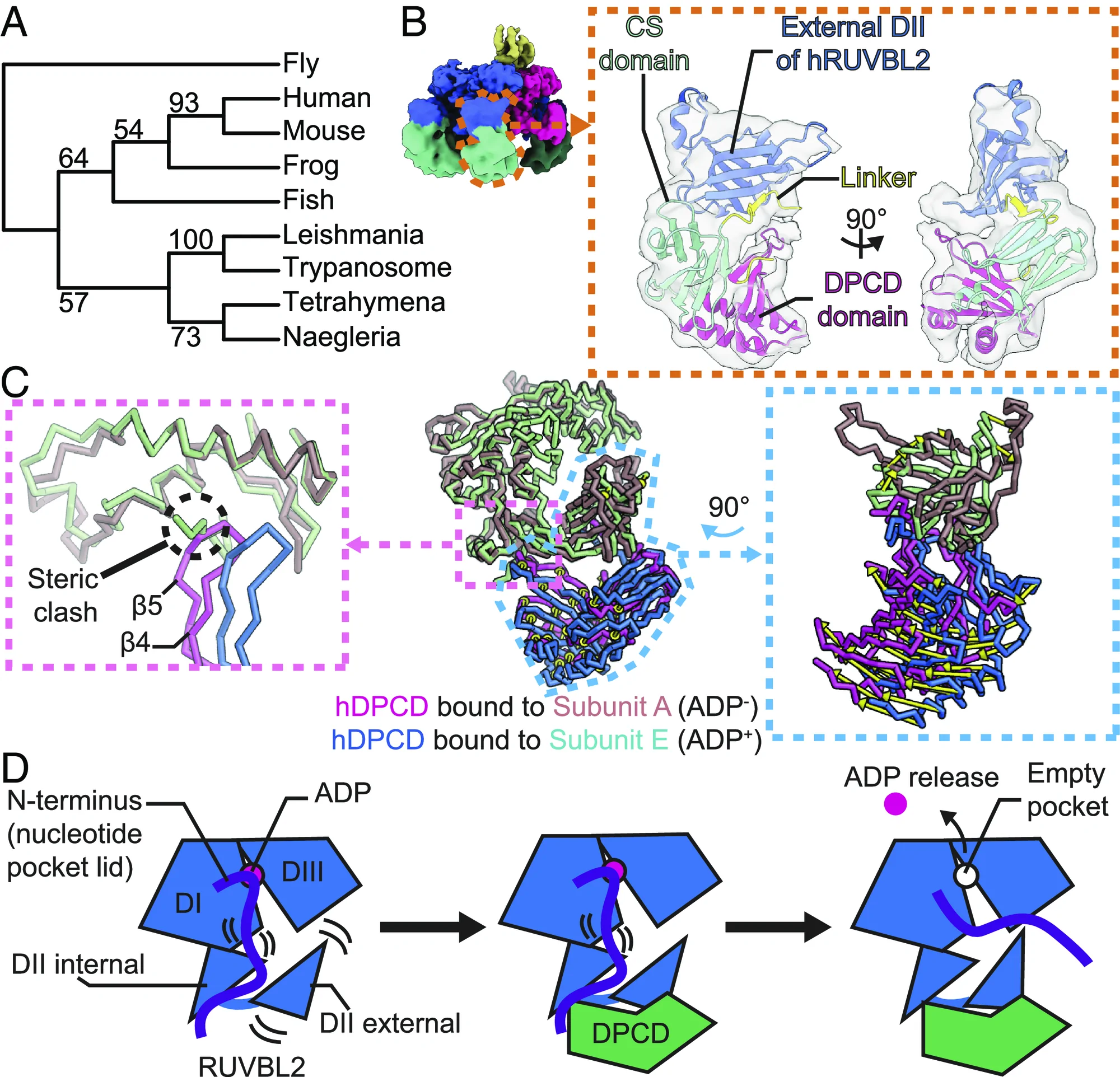
hDNAAF19 and hDPCD form a cocomplex with hRUVBL1/2. (*A*) A phylogenetic tree of DPCD. The numbers are bootstrap values (*n* = 100). (*B*) The structure of hDPCD with the external DII domain of hRUVBL2. The complex is presented in a cartoon format and fitted into the locally refined cryo-EM map of the external DII domain of the subunit A of hRUVBL2 with hDPCD. hRUVBL2 is shown in blue, whereas hDPCD is shown in three different colors: DPCD domain (magenta), CS domain (emerald green), and the linker between these domains (yellow). (*C*) Two different conformations of the external DII domain of hRUVBL2 bound to hDPCD. The Cα traces of subunits A and E of hRUVBL2 bound to hDPCD are shown in a color combination of magenta/salmon pink and blue/cyan, respectively. Pink and cyan dotted squares represent magnified views of the internal and external DII domains of hRUVBL2 bound to hDPCD, respectively. Yellow vectors from subunit E to A were drawn connecting equivalent Cα atoms with a separation distance >6 Å. (*D*) A model of the molecular mechanism of the nucleotide exchange of RUVBL2 by DPCD. Purified RUVBL2 itself has the ADP-bound form, in which the nucleotide-binding pocket gate is closed by the N terminus of RUVBL2. Binding of DPCD in combination with the flexibility of the N terminus of RUVBL2 promotes a conformational change in the N terminus and facilitates the release of ADP from the nucleotide-binding pocket of RUVBL2.

We next analyzed the details of the structure of hDNAAF19–RUVBL1/2–DPCD. DPCD orthologs are present in unicellular and multicellular organisms that have motile cilia (Fig. 7*A*) and are highly conserved among these organisms. The sequence identity between humans and *Leishmania* is ~40%. As was shown before (32, 37), an AlphaFold model of hDPCD revealed that it possesses a core CS domain and a domain unique to DPCD (hereafter referred to as the DPCD domain), which is composed of an N-terminal α-helix and an antiparallel β-sheet, followed by a short α-helix (*SI Appendix*, Fig. S10*A*). Using an AlphaFold model of hDPCD in complex with the external DII domain of hRUVBL2 as a template, the structure of the complex was modeled into the cryo-EM map of hDNAAF19–RUVBL1/2–DPCD. The resolution of the region was not high enough to include the side chains of the model (*SI Appendix*, Fig. S10*B*). The structure showed that the linker between the CS and DPCD domains and the loop between β4 and β5 of hDPCD mainly interacts with the external DII domain of hRUVBL2 (Fig. 7*B*). The linker contains the conserved “NKKYYKKF,” which was previously identified as the key interaction between hDPCD and hRUVBL2 (32).

We next looked at how the interaction between hDPCD and hRUVBL2 affects the structure of hDNAAF19–RUVBL1/2 by comparing the structures of hDNAAF19–RUVBL1/2 and hDNAAF19–RUVBL1/2–DPCD. ADP molecules are present in all six subunits within hRUVBL1 and hRUVBL2 within hDNAAF19–RUVBL1/2, with less occupancies in the subunits A and D of hRUVBL2 (*SI Appendix*, Fig. S7 *A* and *E*). In contrast, ADP molecules were absent and at ~20% occupancy in the subunits A and D of hDNAAF19–RUVBL1/2–DPCD, respectively (*SI Appendix*, Fig. S7 *C* and *E*). Along with this, the densities of the residues before Q44 were not visible in these subunits, while those of the remaining hRUVBL2 subunits in hDNAAF19–RUVBL1/2–DPCD and all the hRUVBL2 subunits in hDNAAF19–RUVBL1/2 were present (*SI Appendix*, Fig. S10*C*). It was shown previously that the hRUVBL2 N terminus, particularly H25 and H27, acts as a gatekeeper for the nucleotide-binding pocket (39). These results imply that hDPCD promotes an open conformation of the N terminus in hRUVBL2 subunits that exhibit incomplete ADP occupancy.

We next investigated the mechanistic basis of how hDPCD regulates the conformation of the hRUVBL2 N terminus. Focused 3D classification targeting hDPCD and the external DII domain of hRUVBL2 revealed that the major classes corresponding to subunits A, B, D, and E display defined density in the masked regions (*SI Appendix*, Fig. S7*C*). Notably, the maps of subunits A and D (ADP−) are different from those of subunits B and E (ADP+). Since the densities of hDPCD in subunits A (ADP−) and E (ADP+) were clearer than those of the other subunits (*SI Appendix*, Fig. S6*A*), we compared whether hDPCD interacted with hRUVBL2 differently in subunits A and E. Their structural comparison showed a rotation movement of the external DII domain of subunit E relative to that of subunit A around the linker connecting the external and internal parts of the DII domain of hRUVBL2 (the light blue dotted box of Fig. 7*C*). Moreover, in subunit A, the loop between β4 and β5 of the hDPCD domain competed with the N terminus of hRUVBL2 for a pocket in the internal DII domain, displacing the N terminus through a latch-and-hook-like mechanism (the light pink dotted box of Fig. 7*C*). In contrast, in subunit E, the loop of hDPCD interacted with a different region of the DIII domain, while the N terminus of hRUVBL2 occupies the pocket (Fig. 7*C*).

Collectively, these observations suggest that hDPCD induces a conformational change in the external DII domain of hRUVBL2 and blocks its N terminus from interacting with a hydrophobic pocket within the internal DII domain of hRUVBL2. This, in turn, shifts the conformation of the N-terminal region of hRUVBL2 to being flexible, opening the gate of the nucleotide-binding pocket and facilitating nucleotide exchange. From our observation, it is likely that the semiflexible conformation of the hRUVBL2 N terminus is a prerequisite (Fig. 7*D*).

## Discussion

We have shown that DNAAF19 plays a role as a DNAAF by interacting with the RUVBL1/2 complex in conjunction with its interacting partners, such as DPCD, in vivo and in vitro.

### Biological Role of the DNAAF19–RUVBL1/2 Complex.

Human DNAAF19 was previously proposed to function as an outer dynein arm docking protein (26). However, our data argue against this model, as hDNAAF19 did not interact with microtubules either in vitro or in vivo and did not localize to axonemes (Fig. 1 *C* and *D*). Instead, our structural and interactome analyses strongly support a role for DNAAF19 in dynein arm assembly through its association with the RUVBL1/2 complex. Indeed, DNAAF19 has been implicated in the dynein arm assembly process and recently renamed DNAAF19 from CCDC103 (40). Unlike other RPAP3-C domain-containing proteins such as RPAP3 and SPAG1, which possess TPR domains that recruit HSP90, DNAAF19 lacks the TPR domain but contains the unique DNAAF19N domain (Fig. 1*A*), which is the only conserved domain in the *Drosophila* DNAAF19 ortholog, suggesting that this region performs an evolutionarily conserved function. We therefore examined whether DNAAF19N might interact with other DNAAFs, but AlphaFold3 modeling of candidate proteins from Fig. 2*C* did not yield reliable interaction predictions (*SI Appendix*, Fig. S11). Nonetheless, we cannot exclude the possibility that DNAAF19N interacts with proteins. Given that *Drosophila* DNAAF19 lacks the RPAP3-C domain, any such interaction would likely regulate the partner protein rather than recruit it to RUVBL1/2. Recent work has reported an interaction between DNAAF19N and the DII domain of RUVBL2 (36), suggesting that DNAAF19N may modulate DII-domain conformation. Because DNAAF19N was not observed in our cryo-EM maps, any such interaction—if present—may be transient, dynamic, or occur only during specific states of the RUVBL1/2 ATPase cycle, such as hexamer assembly or the ATP-bound conformation.

Mass spectrometry pulldown studies of DNAAF19 and its PCD variant H154P in lung and testis lysates showed the interaction between DNAAF19 and RUVBL1/2 and their further association with PAQosome-related proteins including DPCD, PCD proteins including CFAP298, and other cilia-related proteins such as TNPO1. This suggests that RUVBL1/2 plays a role as the protein–protein interaction platform to recruit not only DNAAF19 but also other proteins, including DPCD, as we have shown in our cryo-EM structure (Fig. 5*C*). Transcriptomics shows that *DNAAF19* is markedly upregulated during airway multiciliogenesis similarly to *SPAG1,* another PCD-associated DNAAF, although at lower levels, while *DPCD* behaves more like *RUVBL1/2* in being upregulated during multiciliogenesis but with higher initial levels preciliogenesis, reflecting wider roles. Although the exact biological role of DNAAF19–RUVBL1/2 remains elusive, our results imply that it can form heterogeneous protein complexes that participate in dynein arm assembly. Importantly, our results suggest that the PCD-causing variant H154P impairs the hDNAAF19 interactome by destabilizing the hDNAAF19 protein. The axonemes of PCD patients carrying the H154P variant display diverse phenotypes, ranging from normal to partial loss and total absence of dynein arms, while a loss-of-function *dnaaf19* Q27Stop variant in zebrafish resulted in the absence of dynein arms (23). Collectively, these results suggest that the H154P variant destabilizes hDNAAF19 but retains partial function. Since this variant is unlikely to affect the protein fold of DNAAF19N, the protein instability of the H154P mutant and its weaker interaction with RUVBL2 likely perturbs dynein arm assembly.

Despite being a PCD-associated dynein assembly factor with shared RPAP3-C-like domain architecture, SPAG1 was not detected in lung or testis DNAAF19 pulldowns. However, the lack of direct/indirect copurification could reflect limited detectability, or it may associate with RUVBL1/2 only during specific stages of dynein arm assembly, with transient or stage-restricted interactions underrepresented.

No major structural differences were observed in hRUVBL2 upon hDPCD binding, except within the external DII domain, and no contacts were observed between hDNAAF19 and hDPCD in the hDNAAF19–RUVBL1/2–DPCD complex. These findings suggest that hDNAAF19 and hDPCD bind hRUVBL2 independently, allowing DPCD to associate concurrently with RPAP3 or SPAG1. However, RPAP3 and SPAG1 interact with PIH1D1 and PIH1D2, respectively (29), which compete with DPCD for binding to the internal DII domain of hRUVBL2 (see further discussion below). DPCD may preferentially associate with the DNAAF19–RUVBL1/2 complex. The spatiotemporal regulation of these protein–protein interactions with RUVBL1/2 remains to be elucidated.

### Tissue-Specific Adaptor Usage and a Possible DNAAF19–RPAP3 Heterocomplex in Testis.

The observation that RPAP3 is significantly enriched with DNAAF19 in testis but not lung raises the possibility of tissue-specific R2TP adaptor configurations. Because both RPAP3 and DNAAF19 harbor RPAP3-C-like domains that mainly engage the RUVBL2 DIII face, one model is that testis permits formation of mixed assemblies in which RPAP3- and DNAAF19-containing adaptors are present within the same higher-order complex. An important consideration is whether DNAAF19 and RPAP3 would compete for overlapping binding surfaces on the same RUVBL hexamer, vs. occupying distinct sites or associating within a larger multicomplex assembly. Our cryo-EM structures of hDNAAF19–RUVBL1/2 (Fig. 5*A*) and the RPAP3-C of RPAP3 and SPAG1 (34, 35, 39) support the latter case, suggesting that DNAAF19, RPAP3, and SPAG1 can bind to the same RUVBL1/2 hexamer simultaneously. An alternative explanation is indirect copurification: DNAAF19 robustly enriches RUVBL1/2, and RPAP3 is recovered only in testis because R2TP complexes are more abundant and/or more stably associated with RUVBLs in that tissue. However, if RPAP3 recovery were solely driven by RUVBL copurification, a similar enrichment might be expected in the lung; the absence of significant RPAP3 enrichment in the lung is consistent with tissue-specific differences in complex composition, stability, or relative abundance, although differences in cellular composition and the abundance of actively assembling motile structures between tissues may also contribute.

A further consideration when comparing lung and testis interactomes is the markedly different cellular composition and biological state of these tissues. Adult airway tissue contains a relatively small population of mature multiciliated cells, whereas testis contains large numbers of spermatogenic cells undergoing continuous flagellar assembly. Consequently, proteins recovered in these pulldown experiments may represent a mixture of active assembly intermediates and steady-state protein associations, with the relative contribution of each likely differing between tissues. In addition, equivalent amounts of total tissue lysate were analyzed rather than equivalent numbers of ciliated or flagellated cells, meaning that differences in cell abundance, protein expression levels, or posttranslational regulation may contribute to the tissue-specific interactors identified. Therefore, while proteins enriched in both tissues likely represent core DNAAF19-associated factors, tissue-restricted interactors should be interpreted more cautiously until validated in controlled cellular systems. Future studies using airway epithelial differentiation models will be required to determine whether these interactions reflect direct roles during dynein arm assembly or tissue-specific steady-state associations.

### Asymmetric Stoichiometry of RUVBL1 and RUVBL2 Hexamer.

We found that the predominant stoichiometry of the hRUVBL1:RUVBL2 heterohexamer in copurified hDNAAF19–RUVBL1/2 complex was 1:5. Structural studies of hRUVBL1/2, copurified with and without their interacting proteins from bacterial, insect, and human cells, showed a 3:3 stoichiometry (13). This suggests that RUVBL1/2 prefers a 3:3 stoichiometry. However, RUVBL1 and RUVBL2 are known to form homo-hexamers, and they can have different stoichiometries within their hexamer in vitro (41), allowing for a 1:5 stoichiometry. Our analysis revealed that hRUVBL2 interacts with either hRUVBL1 (3:3 hRPAP3-RUVBL1/2; PDB: 6FO1) or another hRUVBL2 (1:5 hDNAAF19–RUVBL1/2) through well-conserved residues between these proteins (*SI Appendix*, Fig. S12 *A* and *B*). Although the sequences of the last two α-helices of the DIII domain diverge between RUVBL1 and RUVBL2, this variation is unlikely to confer specificity (*SI Appendix*, Fig. S12 *A* and *B*). These results suggest that RUVBL1 and RUVBL2 can form hexamers with variable stoichiometries. Therefore, the equimolar RUVBL1/2 assembly in vivo is likely regulated by factors such as expression levels, binding partners, and/or chaperones.

These findings imply that the coexpression of hDNAAF19 and hRUVBL1/2 in bacteria influences the predominant stoichiometric population of hRUVBL1/2 within the hDNAAF19–RUVBL1/2 complex. While this could represent the result of our experimental conditions, we cannot completely rule out the possibility that RUVBL1/2 hexamers can adopt various stoichiometries and fulfill different roles within cells. Indeed, RUVBL1 and RUVBL2 work independently and/or antagonistically (42–45). Moreover, RUVBL2 has a higher rate of ATPase activity than RUVBL1 (46). The higher copy number of RUVBL2 within the RUVBL1/2 hexamer might confer a higher ATPase activity of the hexamer.

### Nucleotide Exchange of RUVBL2 Is Facilitated by Different Proteins but in a Similar Manner.

Our results suggest that DPCD promotes the release of ADP from the nucleotide-binding pocket of RUVBL2. This correlated with a conformation change of the external DII domain. Similarly, the ADP-empty conformation of RUVBL2 was observed in the subunit interacting with both the RPAP3-C domains of RPAP3 and PIH1D1 (39) and DHX34 (47). Additionally, SPAG1 together with PIH1D2 was shown to promote nucleotide release from RUVBL1/2 (35). These findings suggest that the release of ADP is regulated by a common mechanism by which an adaptor protein of RUVBLs interacts with their internal DII domain, including conformation change of the external DII domain. Unlike PIH1D1, PIH1D2, and DHX34, for all of which the monomer interacts with a RUVBL1/2 hexamer, DPCD interacts with each subunit of RUVBL2, enabling it to specifically regulate the ATP binding/hydrolysis cycle of RUVBL2 independently in the context of the RUVBL1/2 hexamer.

Our results suggest that DPCD is not sufficient to release ADP from the nucleotide-binding pocket of RUVBL2 by inducing a conformational change in its N terminus. It seems that the ADP release requires the N terminus to be partially flexible before DPCD binds to RUVBL2. This is consistent with a recent report showing that DPCD itself did not facilitate ATPase activity of RUVBL1/2 (32). Our cryo-EM structures could not uncover exactly how the N-terminal gate of the nucleotide pocket is opened. Since the absence of ADP was observed in the RUVBL2 subunit that had a clear density of DNAAF19, it might induce a small conformation change that triggers the N-terminus of RUVBL2 to be flexible. This might be caused by the unobserved N-terminal 90 residues of DNAAF19 (Fig. 1*A*). Therefore, DPCD probably needs other proteins to make the N terminus of RUVBL2 flexible. At this moment, we cannot conclude that DNAAF19 and DPCD work in concert to facilitate the nucleotide exchange of RUVBL2. However, our cellular and structural studies imply that they concomitantly play roles in the dynein arm assembly process by interacting with the same RUVBL1/2 hexamer. Thus, we propose DNAAF19–RUVBL1/2–DPCD as a subcomplex of DNAAF assemblies.

In summary, we have demonstrated that DNAAF19 interacts with the RUVBL1/2 hexamer in vitro and in vivo and functions cooperatively with the RUVBLs’ binding partners, such as DPCD, which modulates nucleotide exchange on RUVBL2. Together, our findings indicate that RUVBL1/2 likely serves as a central hub in the ciliary dynein arm assembly pathway, with its ATP hydrolysis cycle playing a critical role in this process. This study contributes an improved molecular understanding of DNAAFs in health and disease, including the critical role of DNAAF19 in dynein assembly and cilia motility and its dysfunction in PCD caused by the H154P variant.

## Materials and Methods

For additional information, please see *SI Appendix*.

### Protein Purification.

Recombinant hDNAAF19 and hDPCD expressed in bacterial cells were purified using Ni affinity purification. hRUVBL1, RUVBL2, and hDNAAF19 coexpressed in bacterial cells were purified using Ni affinity purification followed by size-exclusion chromatography.

### Microtubule-Pelleting Assay.

Microtubule-pelleting assays were carried out as described previously (48).

### SF-TAP Affinity Purification and Proteomic Analysis.

SF-TAP affinity purification of DNAAF19-associated protein complexes was performed as described previously (49), with adaptations for mouse tissue lysates and recombinant bait constructs. The LC–MS/MS workflow is detailed in *SI Appendix*, *SI Materials and Methods*.

### Temporal Gene Expression Profiling During Ciliogenesis in ALI Cultures.

To assess gene expression during ciliogenesis, RNA was extracted from ALI-cultured human epithelial samples and qPCR was performed as detailed in *SI Appendix*, *SI Materials and Methods*.

### Pulldown Assay.

pEGFP-C1-*hDNAAF19*, pEGFP-C1-*hdnaaf19^H154P^*, or pEGFP-C1 was transiently expressed in HEK293T cells. The GFP-tagged proteins were isolated using GFP-Trap beads, and hRUVBL2 was probed using 1/1,000 mouse anti-RUVBL2 [Sigma: SAB4200115-25UL, a kind gift from Prof. Colin Johnson (University of Leeds)].

### Cryo-EM Data and Models.

The cryo-EM data of hDNAAF19–RUVBL1/2 and its complex with DPCD were collected at the Astbury Biostructure Laboratory, University of Leeds. The data processing details are shown in *SI Appendix*, Fig. S6*A* and Table S3. The models of hDNAAF19–RUVBL1/2 and its complex with hDPCD were built using AlphaFold3 (28), Chimera X (50), PHENIX (51), and Coot (52).

### Native Mass Spectrometry.

Purified hDNAAF19 was exchanged into a volatile solution (100 mM ammonium acetate) and was subjected to native mass spectrometry experiments on the Q-Exactive Plus UHMR (Thermo Fisher Scientific). Data were processed and analyzed using MassLynx v.4.1 (Waters) and UniDec (v.4.2.2).

### Circular Dichroism.

The CD spectra of 0.5 mg/mL of purified recombinant hDNAAF19 in 100 mM sodium phosphate buffer, pH 7.4, were acquired on a Chirascan CD Spectrometer (Applied Photophysics). The spectra were analyzed using Pro-Data and Global 3 Analysis software (Applied Photophysics) (53).

## Supplementary Material

Appendix 01 (PDF)

## Data Availability

Cryo-EM maps and protein structure coordinates have been deposited in EMDB (EMD-57214, EMD-57241, EMD-57215, EMD-57217, EMD-57219, EMD-57221, EMD-57234, EMD-57238, and EMD-57239) (54–62) and PDB (29JR and 29KN) (63, 64), respectively.
