# Supplementary material for: DNAAF19–RUVBL1/2 complex recruits multiple adaptors to promote dynein arm assembly
Source: Proc Natl Acad Sci U S A. 2026 Oct 6;123(41):e2612755123. doi: 10.1073/pnas.2612755123 (PMC13663681; doi:10.1073/pnas.2612755123)
Supplement: Supplementary file 1 [file pnas.2612755123.sapp.pdf]

PNAS

Supporting Information for

DNAAF19–RUVBL1/2 complex recruits multiple adaptors to promote dynein arm assembly

Mafalda de Almeida Gomes, Emma Wood, Thomas Burgoyne, Franziska Klose, Denisa Batrinu, Karsten Boldt, Glenn Carrington, Yehuda Halfon, Rachel George, G. Nasir Khan, Tina Beyer, Hannah M. Mitchison, Takashi Ochi

Corresponding authors: Hannah M. Mitchison and Takashi Ochi
Email: h.mitchison@ucl.ac.uk and t.ochi@leeds.ac.uk

This PDF file includes:

Figures S1 to S12
Tables S1 to S4
Supporting Materials
Supporting Materials and Methods
SI References

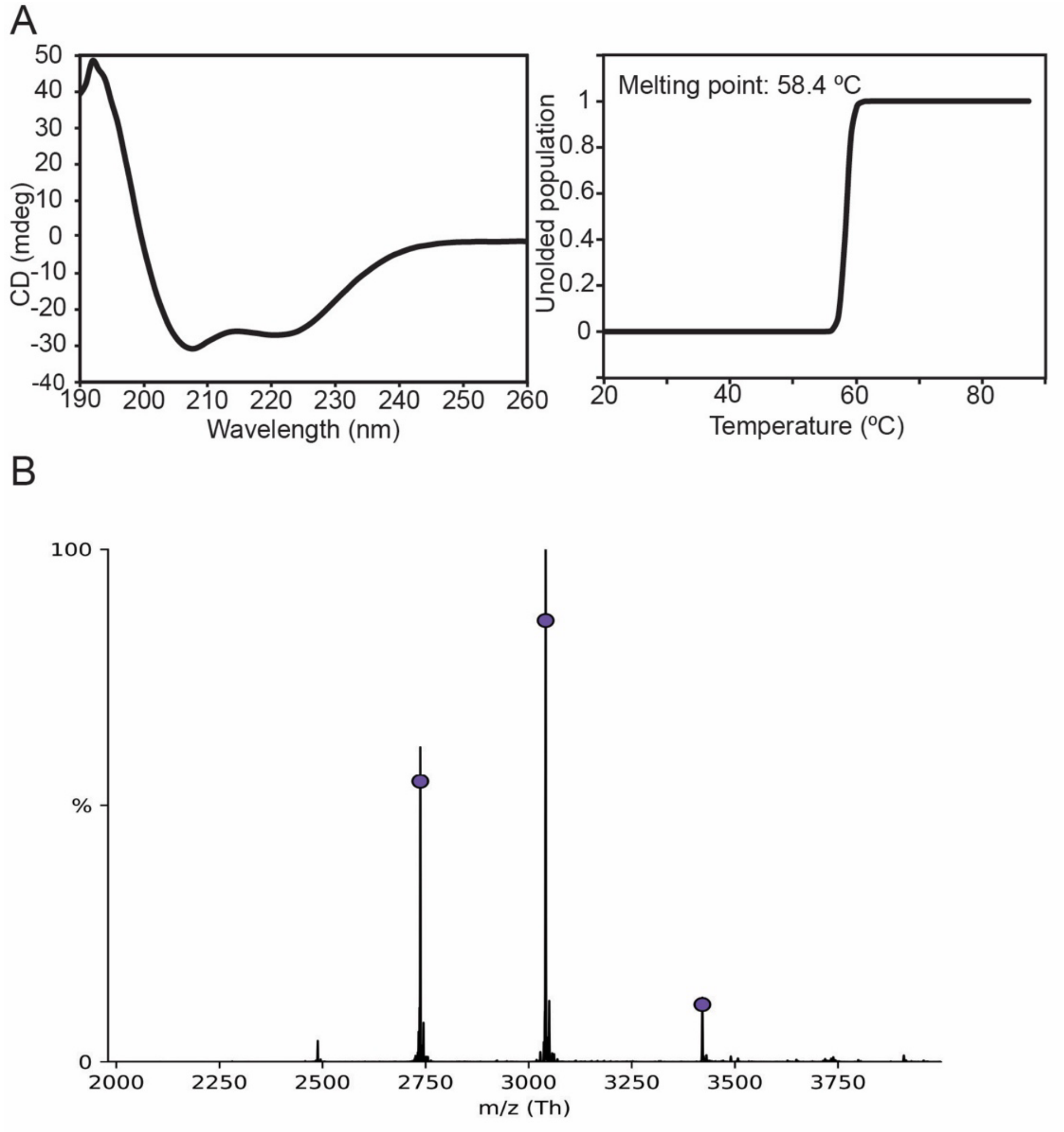

Fig. S1. **Circular dichroism and native mass spectrometric analysis of purified hDNAAF19**.
(A) The CD profile of hDNAAF19. The left panel shows the CD profile of purified hDNAAF19, whereas the right panel shows its melting curve measured at 222 nm. (B) The spectrum displays prominent peaks at approximately m/z 2760, 3000, and 3480 Th (mass-to-charge ratio), each annotated with circles.

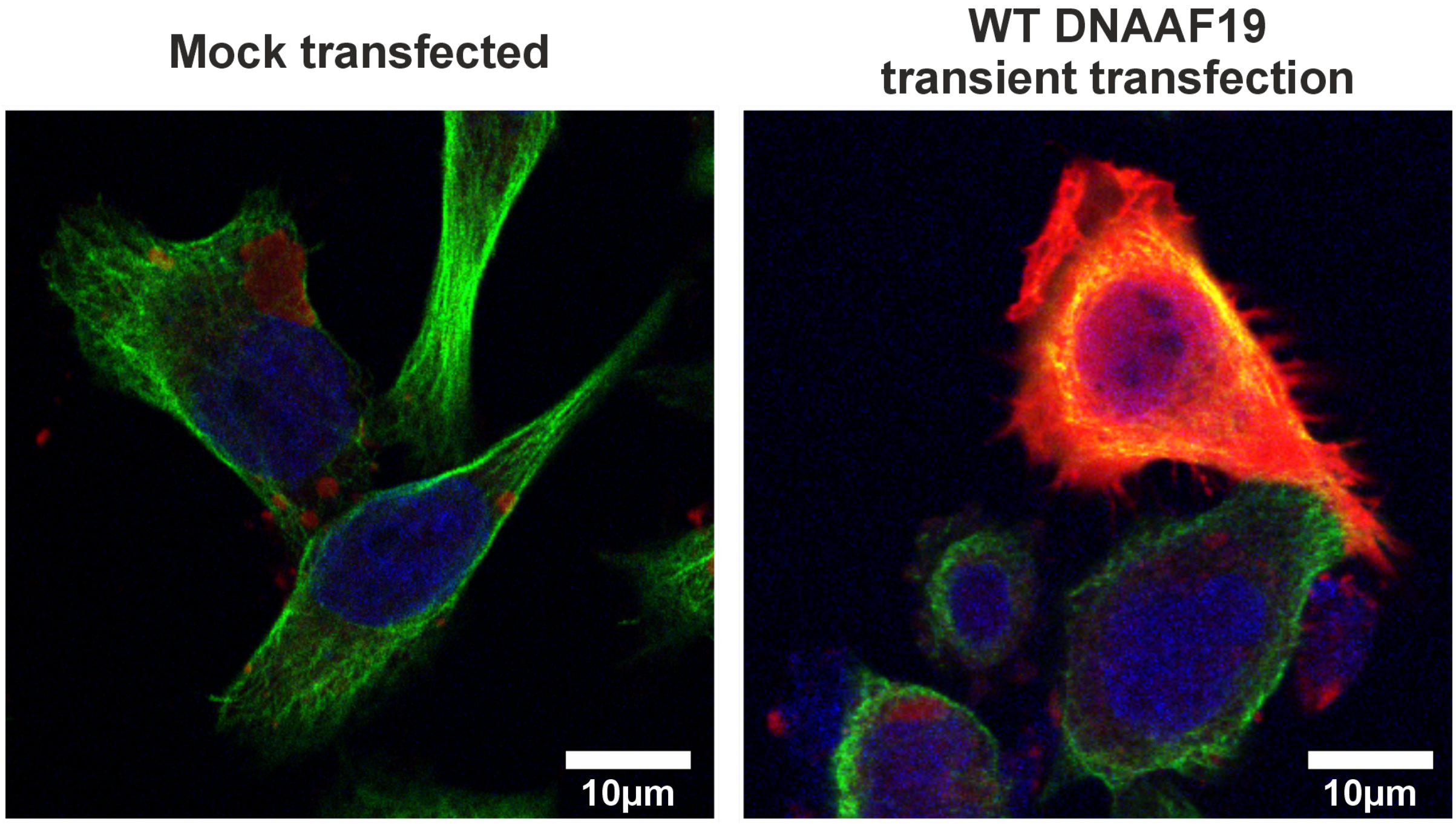

Fig. S2. Validation of DNAAF19 antibody by transient overexpression in HeLa cells. Representative confocal immunofluorescence images of HeLa cells following transient transfection with human DNAAF19 cDNA or mock transfection. Cells were immunostained for DNAAF19 (red), acetylated α-tubulin (green), and nuclei were counterstained with DAPI (blue). Mock transfected cells show minimal endogenous DNAAF19 staining, whereas DNAAF19-transfected cells display a marked increase in cytoplasmic DNAAF19 staining, supporting the specificity of the antibody. Images were acquired using a Leica Stellaris 8 confocal microscope. Scale bars = 10 µm.

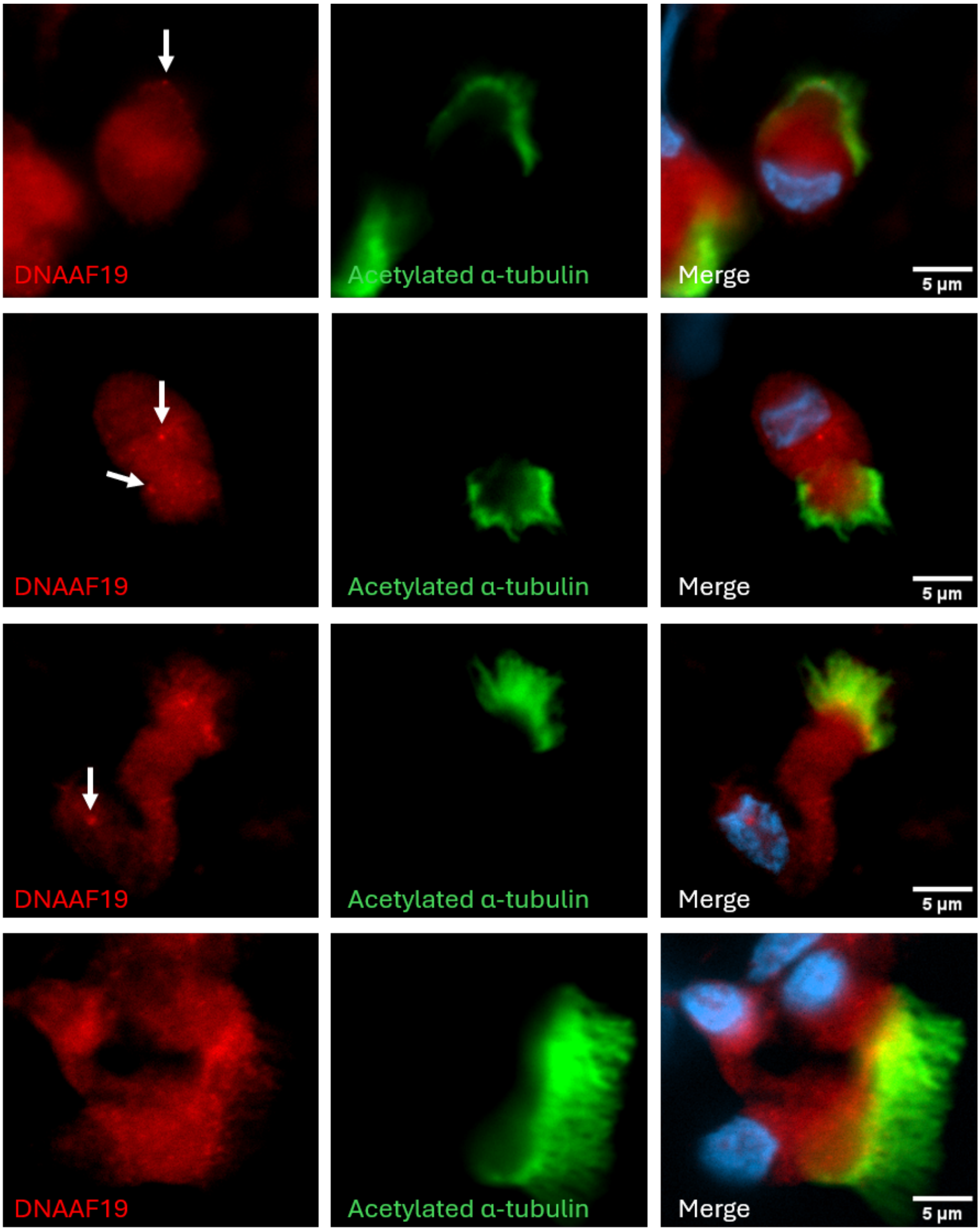

Fig. S3. DNAAF19 localizes to cytoplasmic foci in immature and mature multiciliated human airway epithelial cells.
Representative immunofluorescence images of human airway epithelial cells stained for DNAAF19 (red) and acetylated α-tubulin (green), with nuclei counterstained with DAPI (blue). The top two panels show immature multiciliated cells with short cilia, whereas the bottom two panels show mature multiciliated cells with fully elongated cilia. White arrows indicate representative DNAAF19 cytoplasmic foci, including perinuclear foci in some cells. Images were acquired using a Zeiss Axio Observer 7 widefield fluorescence microscope. Scale bars = 5 µm.

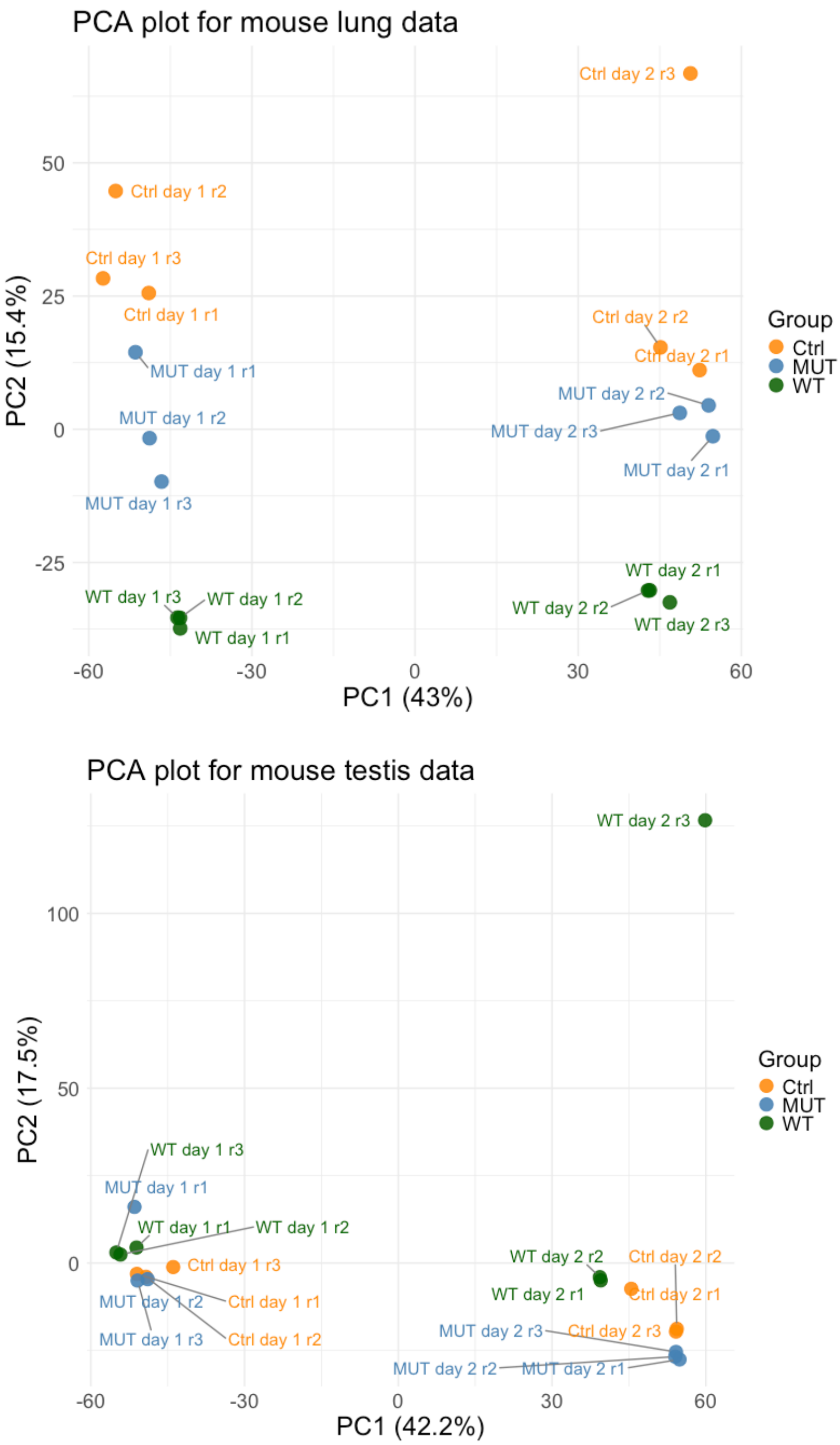

Fig. S4. Principal component analysis of mouse lung and testis proteomic samples. Principal component analysis (PCA) was performed on protein intensity values from mouse lung (**A**) and testis **(B)** samples, including control (Ctrl), wild-type (WT), and *Dnaaf19* H154P mutant (MUT) conditions across two biological days (day 1 and day 2) and three replicates per condition. Each point represents an individual sample, labelled by condition, day, and replicate. PCA was computed using centred and scaled intensity values. The first two principal components (PC1 and PC2) are shown, with the percentage of variance explained indicated on each axis. A major component of the observed variation appears to be associated with the biological day of the experiment.

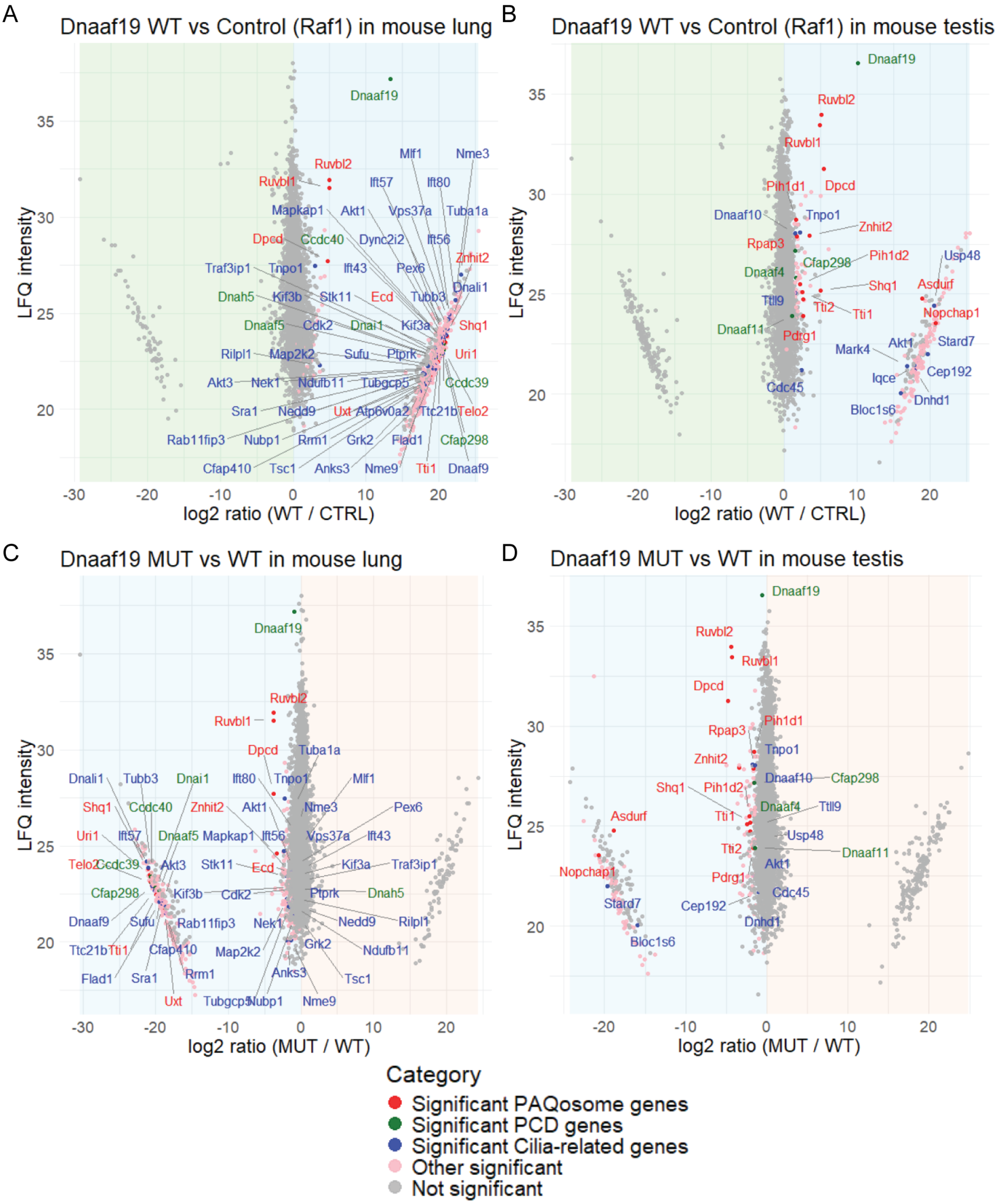

Fig. S5. Dnaaf19 pulldowns highlighting cilia-related protein partners

(A, C) Differential interactome analysis of Dnaaf19 in mouse lung tissue: (A) WT Dnaaf19 versus Raf1 control and (C) H154P mutant (MUT) versus WT comparisons.

(B, D) Differential interactome analysis of Dnaaf19 in mouse testis tissue: (B) WT Dnaaf19 versus Raf1 control and (D) H154P mutant (MUT) versus WT comparisons.

Each point represents a quantified protein based on label-free quantification (LFQ) intensity.

A

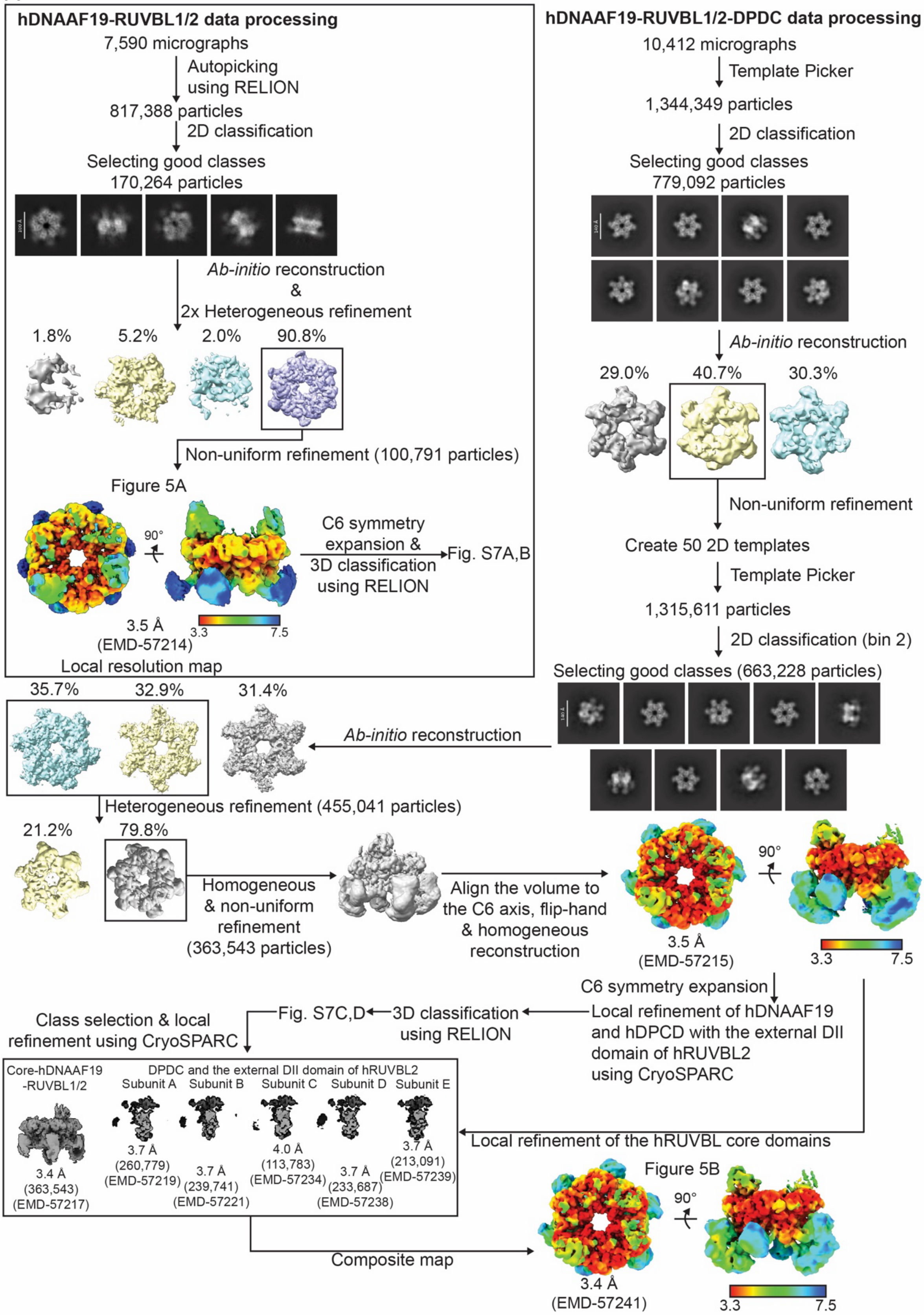
hDNAAF19-RUVBL1/2 data processing
7,590 micrographs
Autopicking using RELION
817,388 particles
2D classification
Selecting good classes
170,264 particles
Ab-initio reconstruction
&
2x Heterogeneous refinement
1.8%
5.2%
2.0%
90.8%
Non-uniform refinement (100,791 particles)
Figure 5A
90°
C6 symmetry expansion & 3D classification using RELION
Fig. S7A,B
3.5 Å
(EMD-57214)
3.3
7.5
Local resolution map
hDNAAF19-RUVBL1/2-DPDC data processing
10,412 micrographs
Template Picker
1,344,349 particles
2D classification
Selecting good classes
779,092 particles
Ab-initio reconstruction
29.0%
40.7%
30.3%
Non-uniform refinement
Create 50 2D templates
Template Picker
1,315,611 particles
2D classification (bin 2)
Selecting good classes (663,228 particles)
Ab-initio reconstruction
35.7%
32.9%
31.4%
Heterogeneous refinement (455,041 particles)
21.2%
79.8%
Homogeneous & non-uniform refinement (363,543 particles)
Align the volume to the C6 axis, flip-hand & homogeneous reconstruction
90°
3.5 Å
(EMD-57215)
3.3
7.5
C6 symmetry expansion
Local refinement of hDNAAF19 and hDPCD with the external DII domain of hRUVBL2 using CryoSPARC
3D classification using RELION
Fig. S7C,D
Class selection & local refinement using CryoSPARC
Core-hDNAAF19 -RUVBL1/2
DPDC and the external DII domain of hRUVBL2
Subunit A
Subunit B
Subunit C
Subunit D
Subunit E
3.4 Å
(363,543)
(EMD-57217)
3.7 Å
(260,779)
(EMD-57219)
3.7 Å
(239,741)
(EMD-57221)
4.0 Å
(113,783)
(EMD-57234)
3.7 Å
(233,687)
(EMD-57238)
3.7 Å
(213,091)
(EMD-57239)
Local refinement of the hRUVBL core domains
Figure 5B
90°
Composite map
3.4 Å
(EMD-57241)
3.3
7.5

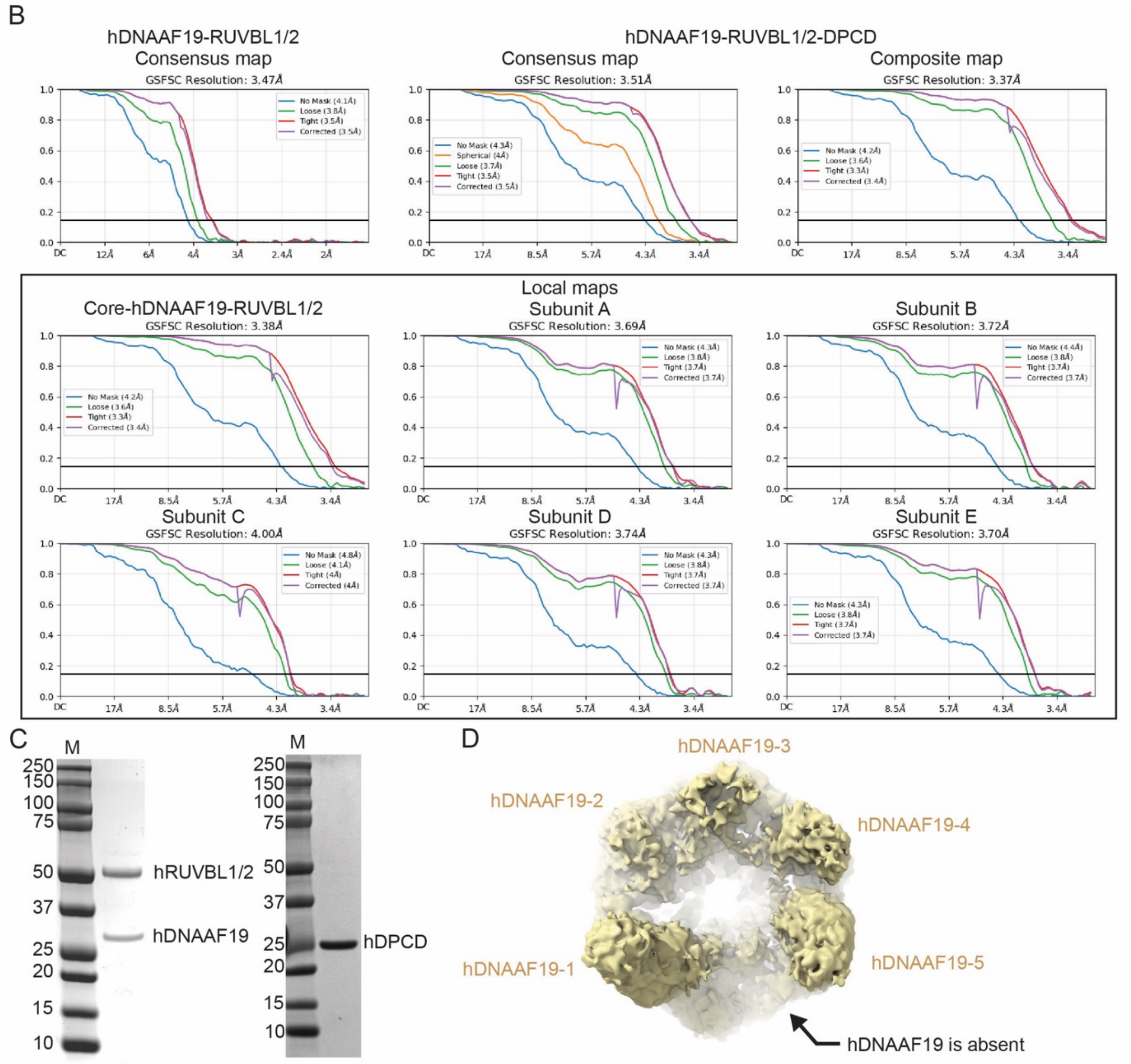

Fig. S6. 1:5 stoichiometry of hRUVBL1/2 complex within hDNAAF19-RUVBL1/2 and hDNAAF19-RUVBL1/2-DPCD complex.

(A) The cryo-EM data processing workflows of hDNAAF19-RUVBL1/2 and its complex with DPCD. The number of particles is shown in parentheses. (B) The FSC curves of deposited maps are shown in (A). (C) An SDS-PAGE gel showing purified hDNAAF19-RUVBL1/2 and hDPCD used for the cryo-EM analyses. (D) The cryo-EM map of hDNAAF19-RUVBL1/2 shown in Figure 5A (main text; the threshold level of 0.12) at a low map threshold level of 0.04.

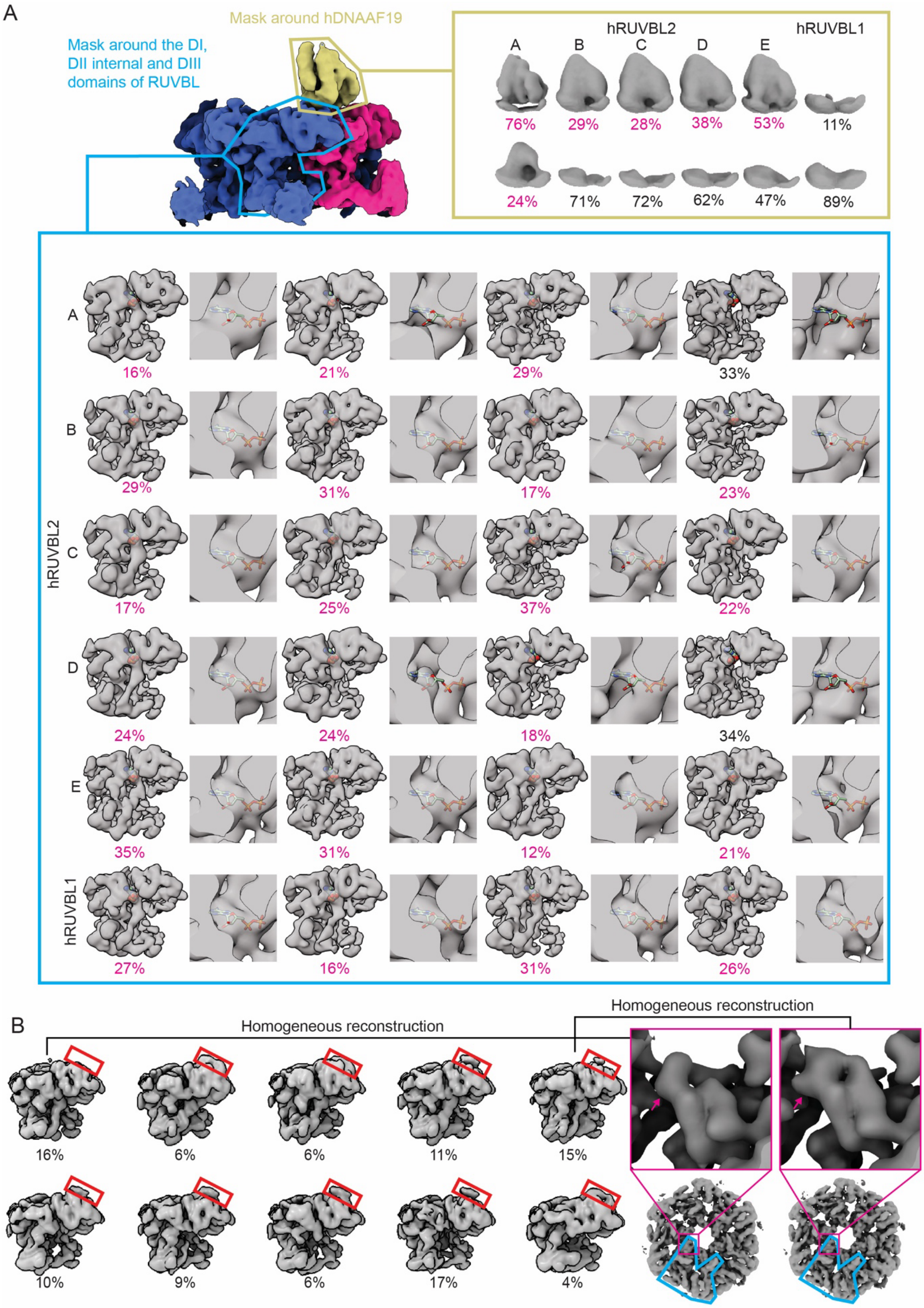
A
Mask around hDNAAF19
Mask around the DI, DII internal and DIII domains of RUVBL
hRUVBL2
A
B
C
D
E
hRUVBL1
76%
29%
28%
38%
53%
11%
24%
71%
72%
62%
47%
89%
hRUVBL2
hRUVBL1
16%
21%
29%
33%
29%
31%
17%
23%
17%
25%
37%
22%
24%
24%
18%
34%
35%
31%
12%
21%
27%
16%
31%
26%
B
Homogeneous reconstruction
Homogeneous reconstruction
16%
6%
6%
11%
15%
10%
9%
6%
17%
4%

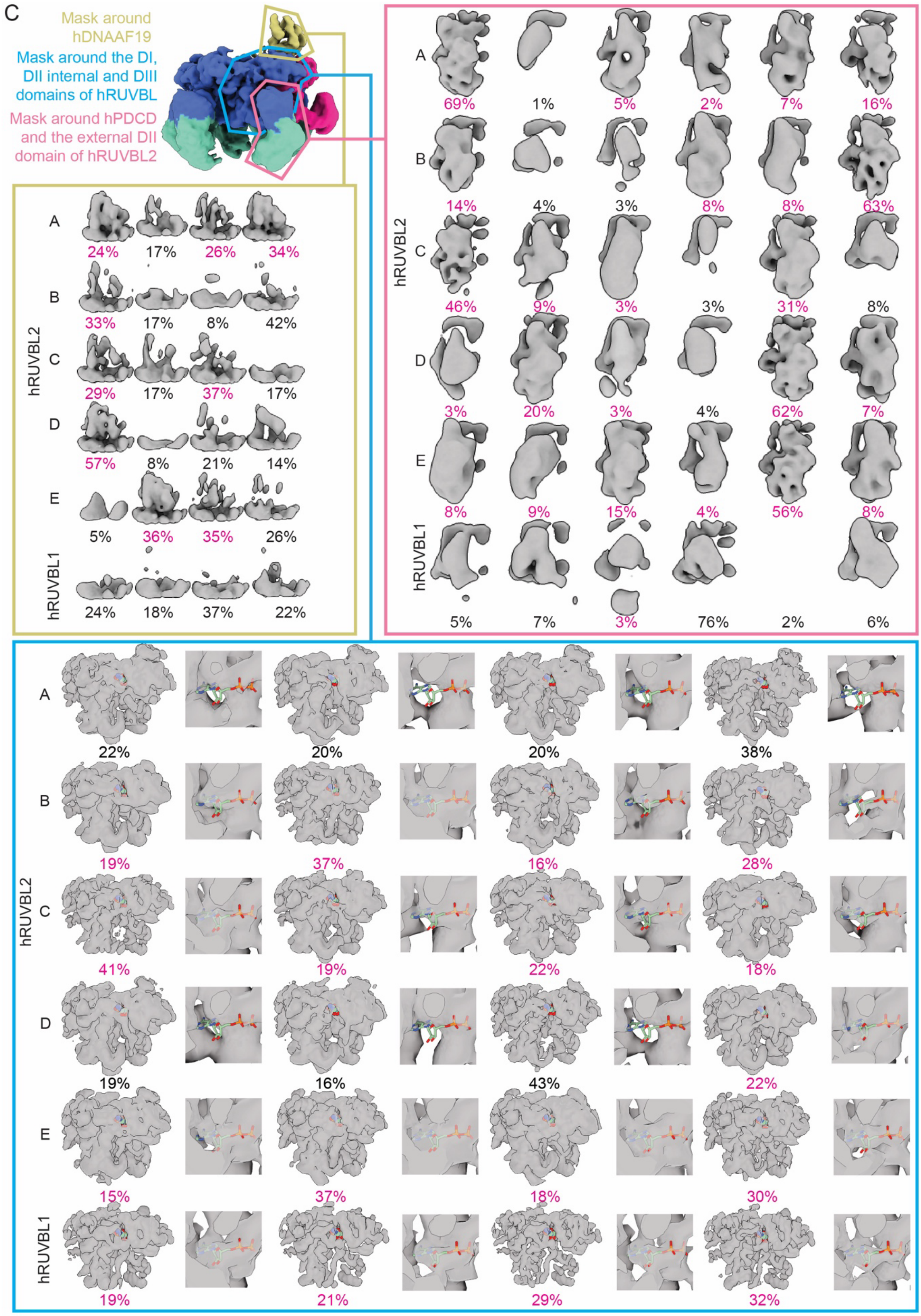
C
Mask around hDNAAF19
Mask around the DI, DII internal and DIII domains of hRUVBL
Mask around hPDCD and the external DII domain of hRUVBL2
hRUVBL2
A
24% 17% 26% 34%
B
33% 17% 8% 42%
C
29% 17% 37% 17%
D
57% 8% 21% 14%
E
5% 36% 35% 26%
hRUVBL1
24% 18% 37% 22%
hRUVBL2
A
69% 1% 5% 2% 7% 16%
B
14% 4% 3% 8% 8% 63%
C
46% 9% 3% 3% 31% 8%
D
3% 20% 3% 4% 62% 7%
E
8% 9% 15% 4% 56% 8%
hRUVBL1
5% 7% 3% 76% 2% 6%
hRUVBL2
A
22% 20% 20% 38%
B
19% 37% 16% 28%
C
41% 19% 22% 18%
D
19% 16% 43% 22%
E
15% 37% 18% 30%
hRUVBL1
19% 21% 29% 32%

D

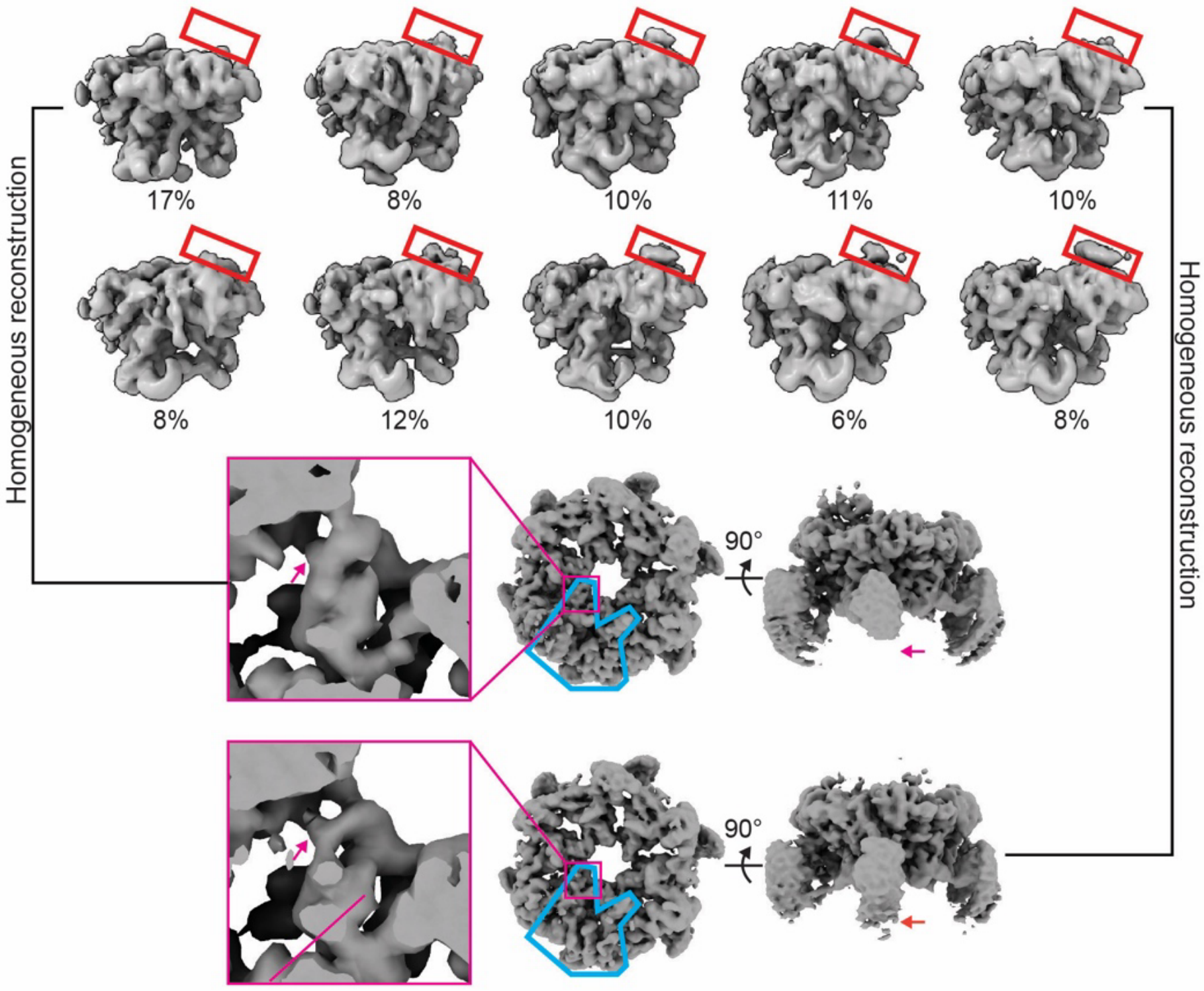

E

Subunit		Occupancy (%)				
		hDNAAF19-RUVBL1/2		hDNAAF19-RUVBL1/2-DPCD		
		hDNAAF19	ADP	hDNAAF19	ADP	hDPCD
hRUVBL2	A	76%	67%	83%	0%	99%
hRUVBL2	B	29%	100%	33%	84%	93%
hRUVBL2	C	28%	100%	66%	100%	89%
hRUVBL2	D	38%	66%	57%	22%	96%
hRUVBL2	E	53%	100%	70%	100%	100%
hRUVBL1		0%	100%	0%	100%	3%

Fig. S7. 3D classification of hDNAAF19-RUVBL1/2 and hDNAAF19-RUVBL1/2-DPCD complex.

(A) Focused 3D classification of the hDNAAF19-RUVBL1/2 complex (EMD-57214; Fig. S6A). The particles used for the complex were subjected to C6 symmetry expansion, and the resulting symmetry-expanded particles were output separately for each symmetry operation to perform 3D classification. The mask regions applied to hDNAAF19 and the RUVBL core are highlighted in dark yellow and light blue, respectively. The resulting classified maps for hDNAAF19 and the RUVBL core are shown in the corresponding-colored boxes. The absence or presence of hDNAAF19 and ADP is indicated by the percentage distributions shown in black and magenta, respectively. (B) Focused 3D classification of the RUVBL core of hDNAAF19-RUVBL1/2 complex. 3D classification was performed on all particles after C6 symmetry expansion. Red rectangles highlight regions with partial hDNAAF19 density. Two classes exhibiting weak hDNAAF19 density were selected for homogeneous reconstruction in cryoSPARC, and the resulting density maps are shown on the right. Magenta arrows indicate the density corresponding to I344 of hRUVBL1 or Y340 of hRUVBL2. (C) Focused 3D classification of the hDNAAF19-RUVBL1/2-DPCD complex (EMD-57215; Fig. S6A). 3D classification was carried out in a similar manner to hDNAAF19-RUVBL1/2. Additionally, focused classification of the region corresponding to hDPCD with the external DII domain of hRUVBL2 (light pink) was performed. (D) Focused 3D classification of the RUVBL core of hDNAAF19-RUVBL1/2-DPCD. 3D classification was carried out in a similar way to hDNAAF19-RUVBL1/2, and the figures presented similarly. Additional magenta arrows

indicate the density region corresponding to hDPCD. (E) Summary of the occupancies of hDNAAF19, hDPCD, and ADP in the hDNAAF19-RUVBL1/2 and hDNAAF19-RUVBL1/2-DPCD complexes, as estimated from the 3D classification results.

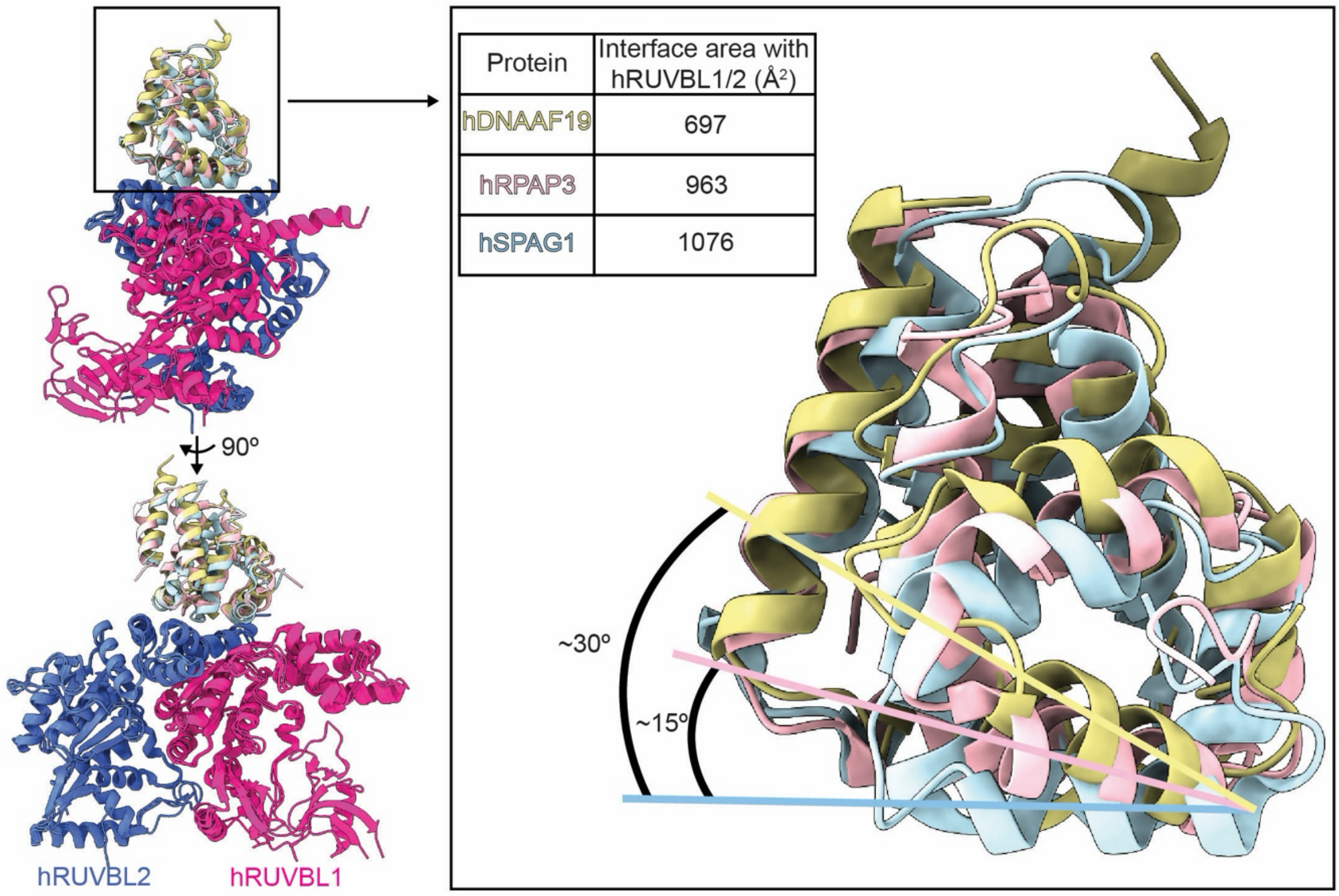

Fig. S8. Structural comparison of the interaction between hRUVBL2 and hDNAAF19 (yellow; hDNAAF19-RUVBL1/2), hRPAP3 (light pink; PDB code: 6FO1) or hSPAG1 (light blue; PDB code: 9HB4).

The interaction interface areas between hRUVBL1/2 and hDNAAF19, hRPAP3 or hSPAG1 were calculated using PDBePISA (1).

A

Coding variant in MANE transcript	Protein change	AlphaMissense	Reference
NM_213607.3:c.104G>C	R35P	Likely pathogenic	(2)
NM_213607.3:c.170_175delinsGG	K57Rfs*121	N/A	(3)
NM_213607.3:c.383dup	P129Sfs*25	N/A	(4)
NM_213607.3:c.461A>C	H154P	Ambiguous	(5)
NM_213607.3:c.548T>C	L183P	Likely pathogenic	(6)
NM_213607.3:c.568_569del	S190Lfs*52	N/A	(7)
NM_213607.3:c.569_570dup	C191Afs*18	N/A	(8)

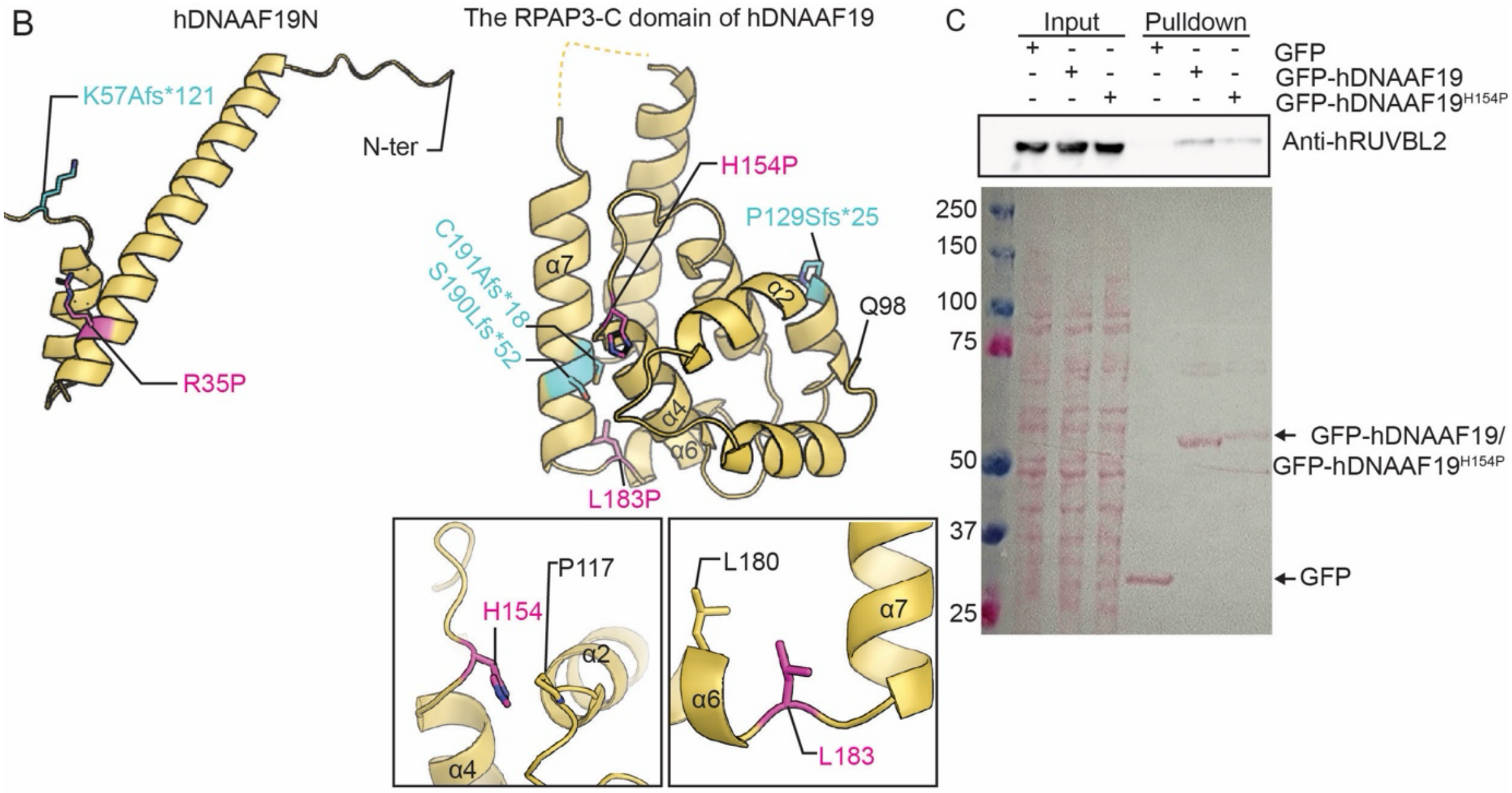

Fig. S9. Known hDNAAF19 variants found in PCD patients.

(A) Summary of the hDNAAF19 PCD-causing variants. (B) An AlphaFold model of the N-terminus of hDNAAF19 (the same model as Fig. 1A) and the RPAP3-C domain of hDNAAF19 (the same structure as Fig. 6) show the location of the PCD-causing variants, with missense changes marked in magenta and frameshift variants in cyan. Q98, P117, and L180 are also labelled to indicate the first residue visible in the hDNAAF19 RPAP3-C structure, and the interacting residues H154 and L183, respectively. (C) hDNAAF19^{H154P} interacts with hRUVBL2. The Western blot and corresponding Ponceau-stained membrane show that both exogenously expressed GFP-hDNAAF19 and its H154P variant interacted with endogenous hRUVBL2. The numbers on the left of the ponceau-stained blot are the molecular weights of protein ladders.

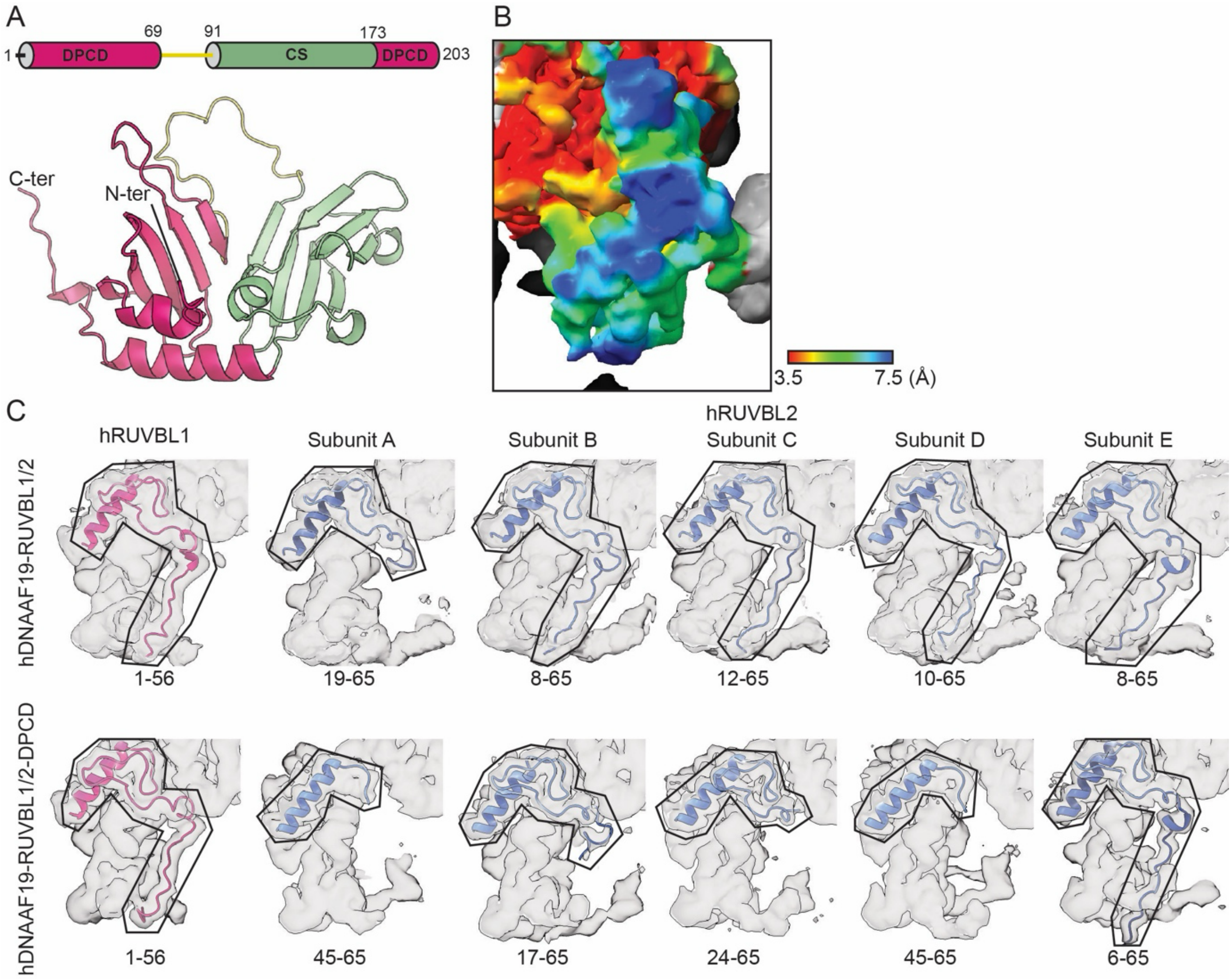

Fig. S10. A hDPCD model and how it affects the structure of hDNAAF19-RUVBL1/2.
(A) The domain architecture and an AlphaFold model of hDPCD. (B) The local resolution map of the region, including hDPCD and the external DII of hRUVBL2 (subunit A). The resolution color scheme is shown on the right of the map. (C) The cryo-EM map regions of the N-termini of hRUVBL1 and hRUVBL2 of hDNAAF19-RUVBL1/2 and its complex with hDPCD. The numbers below the maps indicate the residue ranges of the presented cartoon models. The black lines indicate the N-termini map regions where their models were built.

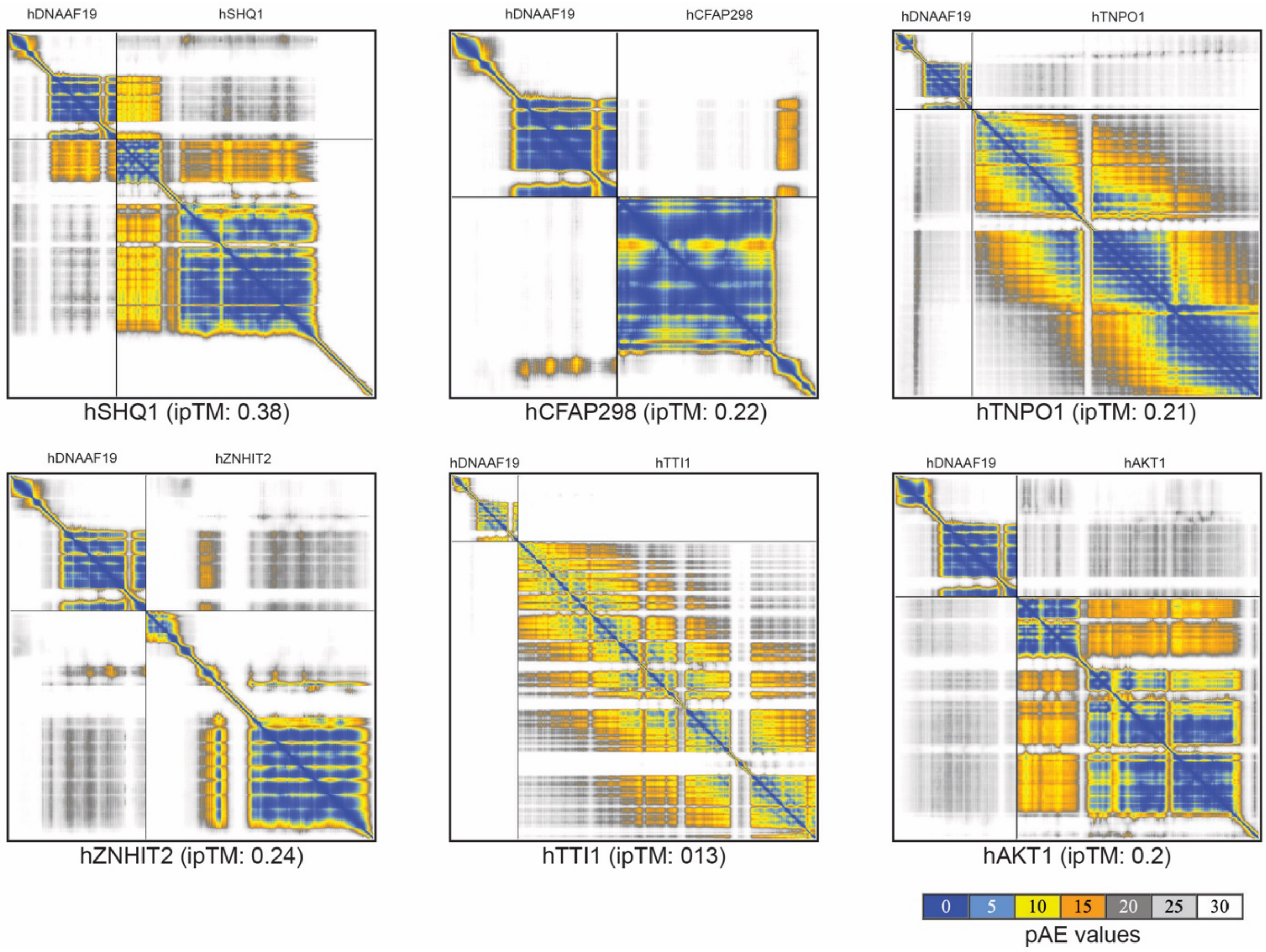

Fig. S11. pAE plots showing the AlphaFold model qualities of hDNAAF19 and its potential interactors.

The selected potential interactors of hDNAAF19 highlighted in bold in Figure 2C were assessed using AlphaFold3. None showed significant interaction with hDNAAF19 based on ipTM scores and pAE plot analyses.

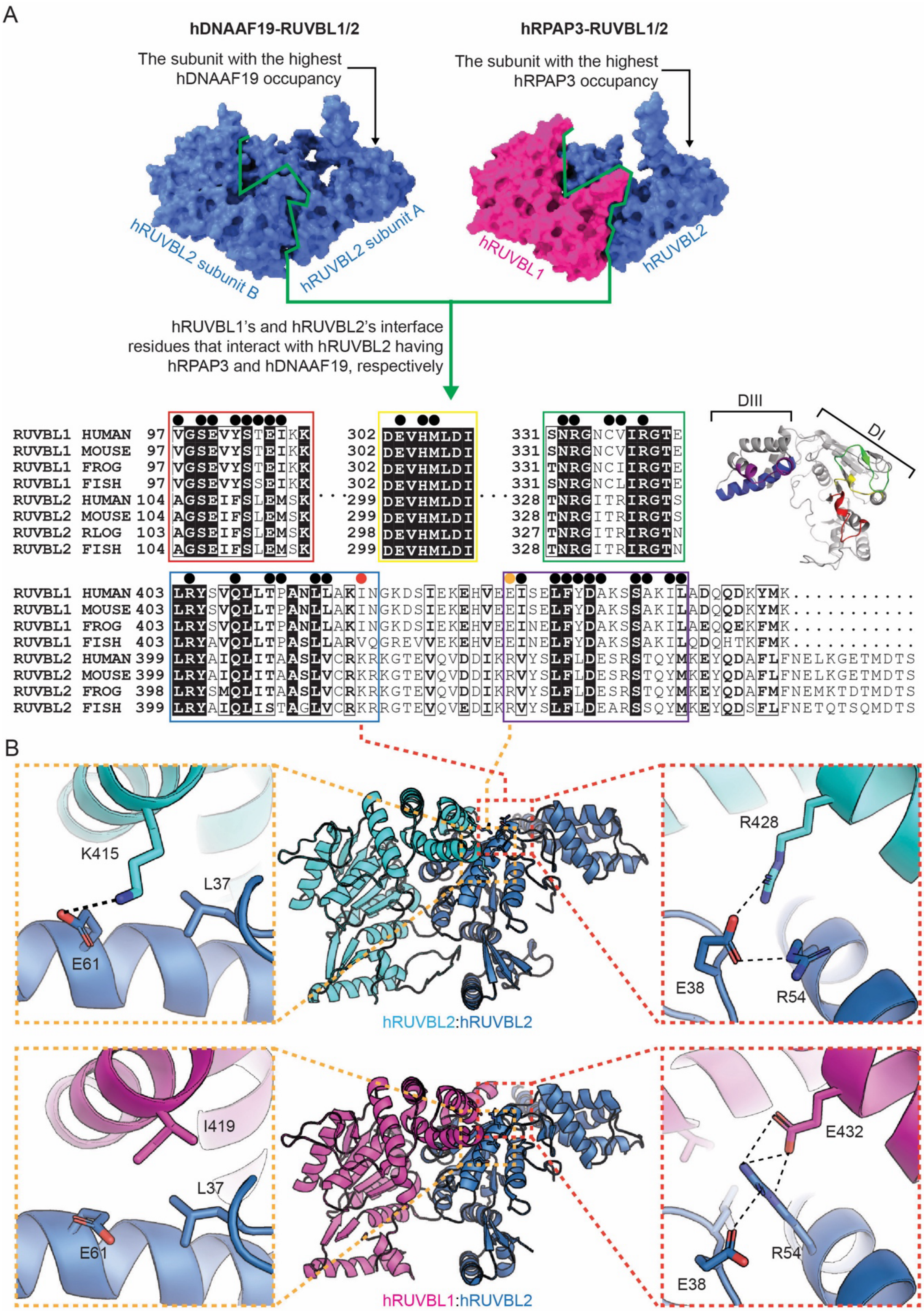

Fig. S12. Key residues involved in the 3:3 and 1:5 RUVBL1/2 hexamers.
(A) Structures of the hRUVBL2 homodimer and the hRUVBL1/2 heterodimer within the hDNAAF19–RUVBL1/2 (our structure) and hRPAP3–RUVBL1/2 (PDB code: 6FO1) complexes, respectively. Dimerization interface residues were analyzed using PDBePISA (1) and PyMOL. A sequence alignment of RUVBL1 and RUVBL2 from human, mouse, frog and

fish are shown below. Five distinct regions that mediate dimerization are highlighted in the alignment and mapped onto the RUVBL structure using five different colors. Black dots above the alignment indicate residues residues that are conserved or have similar physicochemical properties and are involved in dimerization, whereas red and orange dots highlight residues that are not conserved between RUVBL1 and RUVBL2. (B) Structures of the hRUVBL2 homodimer and hRUVBL1/2 heterodimer, highlighting residues marked by red and orange dots. Black dashed lines indicate hydrogen bonds and salt bridges.

Table S1. Selected proteins enriched in wild-type Dnaaf19 pulldown and altered by the H154P mutant in mouse lung

Gene	Category	Reported cilia/cellular compartment	log_2(Dnaaf19 WT/control median intensity)	WT/Control Student's t-test difference	log_2(Dnaaf19 MUT/Dnaaf19 WT median intensity)	H154P MUT/WT Student's t-test difference
Dpcd	PAQosome network	Basal body and axoneme	4.77863	-8.17106	-3.74961	-3.95511
Tti1	PAQosome network	Cytosol and nucleus	19.9025	-19.4674	-19.9025	-19.4674
Telo2	PAQosome network	Cytosol and nucleus	20.676	-17.25	-20.676	-14.1403
Uxt	PAQosome network	Cytosol and nucleus	18.7788	-15.857	-18.7788	-12.6851
Znhit2	PAQosome network	Cytosol and nucleus	21.7224	-15.227	-3.37449	-11.9105
Uri1	PAQosome network	Cytosol	20.8532	-14.224	-20.8532	-10.7854
Shq1	PAQosome network	Cytosol	21.0698	-13.9742	-21.0698	-20.7813
Ecd	PAQosome network	Cytosol	20.3754	-13.5598	-1.02755	-6.9681
Ruvbl2	PAQosome network	Cytosol	5.00235	-4.96204	-3.81555	-3.77634
Ruvbl1	PAQosome network	Cytosol	4.97497	-4.92536	-3.77877	-3.72488
Ccdc39	PCD gene	Axoneme	20.6464	-20.6679	-20.6464	-17.4088
Dnai1	PCD gene	Axoneme	19.9908	-19.8624	-19.9908	-13.3498
Ccdc40	PCD gene	Axoneme	20.6331	-17.2671	-20.6331	-10.3563
Dnah5	PCD gene	Axoneme	19.4558	-13.294	-0.853561	-3.74407
Dnaaf19	PCD gene	Cytosol	13.3365	-22.6513	-0.894969	-0.951433
Cfap298	PCD gene	Cytosol	20.5865	-20.4748	-20.5865	-20.4748
Dnaaf5	PCD gene	Cytosol	19.5272	-9.56989	-19.5272	-13.1643
Dnali1	Cilia-related	Axoneme	20.981	-21.0097	-20.981	-14.3138
Kif3a	Cilia-related	Axoneme	20.1572	-16.9231	-0.758306	-3.71858
Nme9	Cilia-related	Axoneme	17.1343	-16.8055	-1.37437	-5.99639
Ift80	Cilia-related	Axoneme	22.2341	-15.0566	-0.665156	-4.03858
Tuba1a	Cilia-related	Axoneme	23.0051	-15.0032	-0.997838	-7.89093
Ift56	Cilia-related	Axoneme	21.5419	-14.5492	-2.37877	-11.1149
Pex6	Cilia-related	Axoneme	20.6933	-14.1555	-0.985285	-6.73657
Mlf1	Cilia-related	Axoneme	21.4302	-13.6005	-0.197645	-6.52594
Tubb3	Cilia-related	Axoneme	20.7176	-13.5873	-20.7176	-17.2602
Ttc21b	Cilia-related	Axoneme	20.1106	-13.5579	-20.1106	-10.2335
Dync2i2	Cilia-related	Axoneme	20.4545	-13.54	0.0629327	-6.50965
Kif3b	Cilia-related	Axoneme	19.5968	-13.4394	-1.07629	-3.99552
Traf3ip1	Cilia-related	Axoneme	19.4416	-13.3947	-0.202723	-3.53344
Ift57	Cilia-related	Axoneme	21.1914	-10.9688	-21.1914	-10.6479
Ift43	Cilia-related	Axoneme	20.1925	-10.4951	-0.154538	-0.189105
Sra1	Cilia-related	Axoneme	19.0502	-9.77224	-19.0502	-6.79553
Nek1	Cilia-related	Basal body	18.9583	-19.0125	-1.71514	-9.74436
Akt1	Cilia-related	Basal body	20.5352	-13.9764	-1.06728	-4.13995
Rrm1	Cilia-related	Basal body	18.4251	-12.3264	-18.4251	-12.2815

Nedd9	Cilia-related	Basal body	18.0624	-12.1085	-0.113179	-0.098767
Tsc1	Cilia-related	Basal body	17.97	-12.0658	-0.203495	-3.0475
Rilpl1	Cilia-related	Basal body	3.62127	-10.0763	-0.93503	-6.50245
Rab11fip3	Cilia-related	Basal body	18.5894	-9.65946	-18.5894	-9.73094
Cfap410	Cilia-related	Basal body	18.6637	-9.61115	-18.6637	-6.47629
Tnpo1	Cilia-related	Basal body	2.98285	-2.47714	-2.23907	-1.78133
Sufu	Cilia-related	Basal body and axoneme	19.4087	-19.4521	-19.4087	-13.3567
Anks3	Cilia-related	Basal body and axoneme	17.0337	-14.1764	-1.85537	-8.70049
Nubp1	Cilia-related	Basal body and axoneme	17.8212	-11.5411	-0.933006	-6.07248
Stk11	Cilia-related	Basal body and axoneme	20.3034	-10.4953	-1.90984	-7.07301
Tubgcp5	Cilia-related	Basal body and TZ	17.973	-9.38805	-1.70643	-6.38957
Nme3	Cilia-related	Cilia membrane/cilioplasm	22.3216	-15.0663	-0.215369	-0.238683
Mapkap1	Cilia-related	Cilia membrane/cilioplasm	20.5912	-13.8091	-1.51638	-7.00049
Flad1	Cilia-related	Cytosol	19.4582	-19.2618	-19.4582	-19.2618
Map2k2	Cilia-related	Cytosol and axoneme	18.4206	-15.417	-1.92877	-9.3836
Atp6v0a2	Cilia-related	Cytosol	17.504	-14.626	0.359561	0.253316
Vps37a	Cilia-related	Cytosol and basal body	21.2415	-14.6252	-0.426798	-3.75167
Akt3	Cilia-related	Cytosol and basal body	19.0124	-12.7494	-19.0124	-12.6448
Dnaaf9	Cilia-related	Cytosol and axoneme	20.4933	-10.452	-20.4933	-13.7885
Ndufb11	Cilia-related	Cytosol	17.7843	-9.41663	-1.00646	-3.00038
Grk2	Cilia-related	Cytosol and basal body	17.3272	-8.63761	-0.390324	-0.269787
Ptprk	Cilia-related	Membrane	19.2139	-10.2299	-0.39529	-0.4362
Cdk2	Cilia-related	Nucleus	19.508	-16.4126	-0.936082	-6.72804

Table shows selected proteins of interest identified by quantitative mass spectrometry in mouse lung tissue. Differences between conditions were calculated as the subtraction of one condition from the other. WT refers to DNAAF19 wild-type, Control refers to Raf1, and H154P MUT refers to the DNAAF19 H154P missense variant. Protein selection is based on a filtered dataset including only significantly enriched proteins (Student's *t*-test, $p < 0.05$; Significance A, $p < 0.05$). Proteins listed according to category and reported cilia or cellular compartment localization, then within each compartment listed in order of highest difference detected between WT and control. TZ, ciliary transition zone.

Table S2: Selected proteins enriched in wild-type Dnaaf19 pulldown and altered by the H154P mutant in mouse testis

Gene	Category	Reported cilia/cellular compartment	log_2(Dnaaf19 WT/control median intensity)	WT/Control Student's t-test difference	log_2(Dnaaf19 MUT/Dnaaf19 WT median intensity)	H154P MUT/WT Student's t-test difference
Dpcd	PAQosome network	Basal body and axoneme	5.44871	-5.57283	-4.82831	-4.89467
Shq1	PAQosome network	Cytosol	4.99296	-11.6129	-2.05427	-8.19419
Asdurf	PAQosome network	Cytosol and nucleus	18.8729	-11.0719	-18.8729	-14.0425
Tti2	PAQosome network	Cytosol and basal body	2.58784	-8.69658	-2.02134	-1.9593
Pdrg1	PAQosome network	Cytosol and nucleus	2.62522	-5.41179	-1.68491	-1.87041
Ruvbl2	PAQosome network	Cytosol	5.09911	-5.10592	-4.43707	-4.4529
Ruvbl1	PAQosome network	Cytosol	4.9369	-4.94635	-4.28008	-4.30199
Znhit2	PAQosome network	Cytosol and nucleus	3.46982	-3.78104	-3.45483	-3.70163
Tti1	PAQosome network	Cytosol and nucleus	2.49579	-2.4888	-2.4657	-2.42055
Pih1d2	PAQosome network	Cytosol and basal body	2.17826	-2.13262	-2.12087	-2.23433
Rpap3	PAQosome network	Cytosol	1.76457	-1.68767	-1.61713	-1.58771
Pih1d1	PAQosome network	Cytosol and nucleus	1.62151	-1.53105	-1.55068	-1.53704
Nopchap1	PAQosome network	Nucleus	20.7505	-20.8773	-20.7505	-20.8773
Dnaaf19	PCD gene	Cytosol	10.104	-10.529	-0.607661	-0.962638
Dnaaf11	PCD gene	Cytosol	1.10293	-4.20698	-1.49107	-1.44321
Dnaaf4	PCD gene	Cytosol	1.6322	-1.66636	-1.60127	-1.53166
Cfap298	PCD gene	Cytosol and basal body	1.54701	-1.52743	-1.52166	-1.48168
Dnhd1	Cilia-related	Axoneme	17.9519	-12.2055	-0.838243	-3.62346
Iqce	Cilia-related	Basal body	17.8718	-14.7107	0.125043	-5.58949
Usp48	Cilia-related	Basal body	20.5338	-14.036	-0.26364	-3.63071
Akt1	Cilia-related	Basal body	19.3193	-13.0888	-1.52997	-3.90298
Cep192	Cilia-related	Basal body	18.2043	-9.57766	-1.01461	-0.554658
Cdc45	Cilia-related	Basal body	2.35105	-9.23724	-0.588608	-5.98056
Tnpo1	Cilia-related	Basal body	2.13955	-2.04474	-1.7147	-1.62983
Cibar1	Cilia-related	Basal body	-1.52652	9.57106	1.52348	9.64827
Alms1	Cilia-related	Basal body	-20.8981	20.9201	19.0741	10.3398
Mark4	Cilia-related	Basal body and axoneme	16.8015	-9.1174	1.45333	3.35658
Ttll9	Cilia-related	Basal body and axoneme	1.50811	-1.39901	-1.06431	-1.003
Stard7	Cilia-related	Cytosol	19.6441	-13.2781	-19.6441	-13.2781
Dnaaf10	Cilia-related	Cytosol	1.51431	-1.51935	-1.46744	-1.44143
Bloc1s6	Cilia-related	Cytosol and basal body	15.9532	-11.355	-15.9532	-8.7191
Ccdc50	Cilia-related	Cytosol and basal body	-17.96	15.0916	16.937	11.7772
Efcab9	Cilia-related	Membrane	-17.2944	6.2266	16.866	11.835
Slc22a16	Cilia-related	Plasma membrane	-17.2386	11.6471	16.0721	8.7716

Table shows selected proteins of interest identified by quantitative mass spectrometry in mouse testis tissue. Differences between conditions were calculated as the subtraction of one condition from the other. WT refers to DNAAF19 wild-type, Control refers to Raf1, and H154P MUT

refers to the DNAAF19 H154P missense variant. Protein selection is based on a filtered dataset including only significantly enriched proteins (Student's *t*-test, $p < 0.05$; Significance A, $p < 0.05$). Proteins listed according to category and reported cilia or cellular compartment localization, then within each compartment listed in order of highest difference detected between WT and control.

Table S3. Cryo-EM data collection parameters.

**Dataset**	**hRUVBL1/2-DNAAF19**	**hRUVBL1/2-DNAAF19-DPCD**
**Microscope**	Krios 2	Krios 1
**Detector**	Falcon 4i (counting)	Falcon 4i (counting)
**Magnification**	96 k	165 k
**Pixel size (Å)**	0.85	0.74
**Micrographs**		
**Dose per pixel/second**	8.04	7.23
**Dose perÅ2/second**	11.12	13.20
**Exposure time (sec)**	4.51	3.83
**Defocus range (µm)**	-0.9 to 3.0	-0.9 to 3.0
**Total dose**	50.18	50.56
**Dose per frame**	1.003	1.136
**Frames**	50	44

Table S4. Atomic models of hDNAAF19-RUVBL1/2 and hDNAAF19-RUVBL1/2-DPCD.

Dataset	hDNAAF19-RUVBL1/2	hDNAAF19-RUVBL1/2-DPCD
Structure	hDNAAF19-RUVBL1/2	hDNAAF19-RUVBL1/2-DPCD
EMDB codes	EMD-57214	EMD-57241, EMD-57215, EMD-57217, EMD-57219, EMD-57221, EMD-57234, EMD-57238, EMD-57239
PDB codes	29JR	29KN
Map		
Pixel size (Å)	0.85	1.48
Resolution (Å) (0.143 FSC threshold)	3.47	3.51
Sharpening B factor (Å^2)	-146.4	-155.7
Model Refinement		
Initial model		
hDNAAF19-RUVBL1/RUVBL2 1:5 complex	AlphaFold 3 generated	hDNAAF19-RUVBL1/2
hDPCD		AlphaFold 3 generated
Model resolution (Å) (0.5 FSC threshold)	3.8	3.7
Model map correlation	0.85	0.79
Model composition		
Non-hydrogen atoms	15967	21636
Protein residues	2111	3370
Ligands	6	4
Protein mean B factors (Å2)	213.74	197.09
R.M.S.D.		
Bond lengths (Å)	0.01	0.009
Bond angles (°)	1.223	1.473
Validation		
MolProbity score	2.13	2.53
Clashscore	10.68	24.75
Poor rotamers (%)	2.64	4.02
Ramachandran plot		
Favoured (%)	96.16	96.86
Allowed (%)	3.84	3.14
Disallowed (%)	0	0

Supporting Materials

Primers used for qPCR analysis

Gene Target	Forward (5' to 3') sequence	Reverse (3' to 5') sequence
DNAAF19	GGAGAATGCTGCCAAGTTACGG	TATCCTTCCGCTCCAGTGGCTT
SPAG1	GCAGATAGACTGTGGACTCCAG	GCAGGAATAGGTGACAGCTTCTC
RUVBL1	GAAGACAGAGGTGCTGATGGAG	CTCTGTCTCACACGGAGTTAGC
RUVBL2	GGTCTACTCACTCTTCCTGGAC	GGATGACATCCAACTCAGGAGG
DPCD	CAGACGGCAAGGAAATGGCTGA	GGTCTCCTACTTCAAGCTGCCA
GAPDH	GTCTCCTCTGACTTCAACAGCG	ACCACCCTGTTGCTGTAGCCAA

Antibodies used for immunofluorescence staining

Name/immunogen	Conjugate	Host Species	Supplier, Catalogue No.
**Primary antibodies**			
DNAAF19 (CCDC103, 1-242AA)		Rabbit	PA5-113107 ThermoFisher
α-Tubulin monoclonal		Mouse	T6793 Merck
**Secondary antibodies**			
anti-Mouse IgG (H+L)	Alexa Fluor 488	Donkey	A-21202 ThermoFisher
anti-Rabbit IgG (H+L)	Alexa Fluor 555	Donkey	A-31572 ThermoFisher

Curated gene lists used for annotation of scatter plots. Gene lists included:

(i) PAQosome components (9–11): Aar2, Asdurf, Dpcd, Ecd, Hop, Hsp90, Hsp90aa1, Hsp90ab1, Naf1, Nopchap1, Nufip1, p23, Pdrg1, Pfdn6, Pih1d1, Pih1d2, Polr2e, Ppp2r5e, Rpap3, Rpb5, Ruvbl1, Ruvbl2, Shq1, Telo2, Tti1, Tti2, Uri1, Uxt, Wdr92, Znhit2, Znhit3, Znhit6.

(ii) PCD-associated genes (12, 13): Armc4, C21orf59, Ccdc114, Ccdc151, Ccdc164, Ccdc39, Ccdc40, Ccdc65, Ccno, Cep290, Cfap221, Cfap298, Cfap300, Cfap46, Cfap54, Cfap57, Cfap74, Clxn, Dnaaf1, Dnaaf11, Dnaaf19, Dnaaf2, Dnaaf3, Dnaaf4, Dnaaf5, Dnaaf6, Dnaaf7, Dnah1, Dnah10, Dnah11, Dnah5, Dnah7, Dnah9, Dnai1, Dnai2, Dnajb13, Dnal1, Drc1, Drc2, Drc4, Dyx1c1, Foxj1, Gas2l2, Gas8, Heatr2, Ift74, Lrrc56, Lrrc6, Mcidas, Nek10, Nme5, Nme8, Odad1, Odad2, Odad3, Odad4, Ofd1, Pih1d3, Rpgr, Rsph1, Rsph3, Rsph4a, Rsph9, Spag1, Stk36, Ttc12, Zmynd10.

(iii) >14,000 cilia-related genes (not individually listed) extracted from Gene Ontology (GO) terms, including *cilium, motile cilium, motile cilium assembly, cilium movement, ciliary basal body, ciliary base, ciliary transition zone,* and *intraciliary transport*, using the GO database (version 2.5.17).

Supporting Materials and Methods

Constructs

The human *DNAAF19* gene and its H154P mutant were amplified from pET16b-*hDNAAF19* (5) and cloned into a lipoyl-domain-tagged *E. coli* expression vector or pEGFP-C1 vector. The open reading frames of human *RUVBL1*, *RUVBL2,* and h*DPCD* were amplified from a human cDNA library derived from TERT-RPE1 cells (14). The h*RUVBL1*, h*RUVBL2,* and h*DNAAF19* genes, fused to the lipoyl-domain gene, were assembled into a single pHAT5 vector to co-express them. The h*DPCD* gene was cloned into the pHAT4 vector (15).

Protein purification

Lipoyl-tagged hDNAAF19 was expressed at 16 ºC in OverExpress C41(DE3) cells (Cambridge Bioscience) overnight. The pelleted cells were suspended in 50 mM Tris-HCl, pH 7.4, 300 mM NaCl, 10 mM imidazole, 2 mM β-mercaptoethanol, and 1 mM AEBSF, and lysed by sonication. Cell debris was removed by centrifugation, and the supernatant was mixed with 5 ml of HisPur Ni-NTA resin (Thermo Fisher Scientific) on a rotating platform for 90 min at 4 ºC. The resins were washed with the same lysis buffer but with 30 mM imidazole, and bound proteins were eluted with this buffer with 300 mM imidazole. TEV protease was added to the elution, which was dialysed against 2 L of 20 mM Tris-HCl, pH 7.4, 150 mM NaCl, 2 mM β-mercaptoethanol at 4 ºC overnight. The dialysed sample supplied with 20 mM imidazole was mixed with 5 ml of HisPur Ni-NTA resin on a rotating platform for 90 min at 4 ºC, and the supernatant was collected. It was diluted 3-fold with 20 mM Tris-HCl, pH 7.4, 2 mM DTT, and loaded onto a 5 ml HiTrap Q HP column (Cytiva), equilibrated with 20 mM Tris-HCl, pH 7.4, 50 mM NaCl, 2 mM DTT. The bound proteins were eluted with a linear gradient of 20 mM Tris-HCl (pH 7.4), 500 mM NaCl, and 2 mM DTT. The peak containing DNAAF19 was collected, concentrated, and snap-frozen in liquid nitrogen for storage at -80 ºC.

pHAT5-h*RUVBL1/2*-Lipo*DNAAF19* was transformed into OverExpress C41(DE3) cells and expressed and purified using Ni-NTA as described above. The elution was dialyzed against 2 L of 20 mM Tris-HCl, pH 7.4, 200 mM NaCl, 2 mM 2-mercaptoethanol, 10% (v/v) glycerol after TEV was added. 4 ml of Ni-NTA beads were added to RUVBL1/2-DNAAF19 after removing precipitants, and the mixture was incubated for 60 min at 4 °C. The supernatant was collected, concentrated to ~0.5 ml, and loaded onto a Superdex 200 10/300 increase column equilibrated with 20 mM HEPES, pH 7.5, 250 mM NaCl, 2% (v/v) glycerol, 2 mM DTT. The first peak fractions were collected, concentrated, and snap-frozen in liquid nitrogen for storage at -80 ºC.

pHAT4-*hDPCD* was transformed to BL21(DE3) cells (New England Biolabs), and DPCD was purified similarly to DNAAF19 until the second Ni-NTA step. The elution was buffer-exchanged to 20 mM Tris-HCl, pH 7.4, 200 mM NaCl, 2 mM DTT, concentrated, and snap-frozen in liquid nitrogen for storage at -80 ºC.

Microtubule pelleting assay

Tubulin was isolated from porcine brains using the protocol available on Prof Anthony Hyman's website. Microtubule pelleting assays were carried out as described previously (14). 20 µM taxol-stabilized microtubules were incubated with an equal molar amount of DNAAF19 in BRB80 buffer (80 mM PIPES pH 6.8, 1 mM EGTA, 1 mM MgCl2) supplied with 20 µM taxol and 1 mM DTT for 15 min at room temperature. The samples were centrifuged for 30 min at 70,000 rpm at 25 ºC. Their supernatant and pellet fractions were analysed using SDS-PAGE.

SF-TAP affinity purification and proteomic analysis

Wild-type (WT) and H154P mutant mouse Dnaaf19 fusion proteins were expressed in HEK293 cells, purified by FLAG-affinity purification and used to capture interacting proteins from mouse lung and testis lysates, followed by quantitative mass spectrometry. Affinity-purified eluates were precipitated and digested with trypsin. Tryptic peptides from bulk proteomics and miniTurbo proximity labelling were processed using a label-free high-resolution LC-MS/MS approach using data-independent acquisition mode (DIA) on an Orbitrap Tribid Fusion mass spectrometer as previously described (16). MS raw data files of the samples were analyzed using DIA-NN 1.9.2 (17) in library-free mode against the mouse database (Swiss-Prot, #17,283 proteins). High-accuracy spectra with a minimum precursor FDR of 0.01 and tryptic peptides were used for protein abundance label-free quantification. LFQ intensity values were normalised and statistically analysed in Perseus (v1.6.5.0). Proteins identified by reverse sequence, site only, or contaminants were excluded. Only proteins with ≥ 1 unique peptide and present in ≥ 4/6 of replicates per group were retained.

Significance A (Benjamini–Hochberg FDR < 0.05) and Student's *t*-test ($p < 0.05$) were used to identify significantly enriched proteins. Differential abundance analyses compared WT versus control, and WT versus H154P mutant pulldowns, separately for lung and testis samples.

For data visualization, scatter plots were annotated using curated gene sets representing (i) known PAQosome components, (ii) genes implicated in PCD, and (iii) cilia-related genes. PAQosome and PCD gene sets were manually curated from the literature, while cilia-related genes were defined by Gene Ontology annotations associated with ciliary structure and function (GO database version 2.5.17). Gene lists are provided in this SI Appendix with supporting references. Proteins significantly altered in both comparisons were subjected to GO Cellular Component enrichment analysis using ShinyGO (v0.80). All data used in this paper are deposited at MassIVE (massive.ucsd.edu).

Air-liquid interface (ALI) culture of human nasal epithelial cells

Human nasal epithelial cells from a control healthy donor were transduced with BMI-1 for immortalisation of the cells, as described (18). BMI-1 immortalised basal cells were expanded in PneumaCult™-Ex Basal medium (Stemcell Technologies) and seeded in Transwell inserts (Corning, 0.4 µm pore size, 24-well format) pre-coated with 1% collagen in PBS. Cells were seeded onto the inserts at a density of 350,000 cells per insert in 250 µl complete PneumaCult™-Ex Basal medium (apical) with 500 µL in the basal chamber. After 24 hours, the apical medium was aspirated to expose the cell layer to air, establishing the ALI. Cells were then fed only from the basal side with 500 µL PneumaCult™-ALI Basal Medium (Stemcell Technologies) supplemented according to the manufacturer's guidelines, replaced every 2-3 days.

Temporal gene expression profiling during ciliogenesis in air-liquid interface (ALI) cultures

Human airway epithelial samples collected with informed consent (Living Airway Biobank, Northwest Liverpool East REC 19/NW/0171; IRAS ID:261511 or London-Bloomsbury REC 08/H0713/82; IRAS ID 103488) were grown under standard BMI1/ALI culture conditions. To assess gene expression during ciliogenesis, RNA was extracted using the ReliaPrep RNA Cell Miniprep System (Promega) from ALI culture epithelial samples at five time points (day 0, pre-airlift, and days 7, 14, 21 and 28 post-airlift). cDNA was synthesised using the High-Capacity RNA-to-cDNA Kit (Thermo Scientific), followed by qPCR for *DNAAF19, SPAG1, RUVBL1, RUVBL2, DPCD*, normalised to *GAPDH*; however, as *GAPDH* Ct values varied across ALI differentiation timepoints, ΔΔCt-derived fold changes are interpreted as relative temporal trends rather than absolute longitudinal expression changes. The experiment included three technical replicates and three biological replicates per condition and gene target.

Immunofluorescence staining

Control airway epithelial cells were fixed in 4% paraformaldehyde (PFA) in PBS for 20 min before embedding in optimal cutting temperature (OCT) compound at -78°C. For cryo-sections, 15 µm thick sections were cut using a cryostat and placed onto glass slides. For nasal brush samples, respiratory epithelial cells obtained by nasal brush biopsy were suspended in cell culture medium, air-dried onto glass slides, and stored at -80°C until use. Cryo-sections and nasal brush samples were stained with antibodies against DNAAF19 (1:100 PA5-113107 ThermoFisher Scientific) and acetylated α-tubulin (1:200 T6793 Merck) for 2 h at room temperature before incubating with secondary antibodies (Supplementary Materials) for 1 h at room temperature. Coverslips were mounted using ProLong Gold Antifade Mountant with DAPI (11549306 ThermoFisher Scientific). Images of cryo-sections were acquired on a Leica Stellaris 8 confocal microscope, while nasal brush samples were imaged using a Zeiss Axio Observer 7 widefield fluorescence microscope.

Transient overexpression of DNAAF19 in HeLa cells

HeLa cells cultured on glass coverslips were transfected with human *DNAAF19* cDNA cloned into a pcDNA3.1 backbone using Lipofectamine 3000 reagent (Invitrogen) for 24 hours following the manufacturer's guidelines. The cells were fixed in 4% PFA in PBS for 20 mins at room temperature (RT). Subsequently, the cells were permeabilized with 0.2% saponin in PBS for 20 min at RT before incubating in blocking solution containing 0.02% saponin, 1% BSA in PBS for 30 min at RT. Antibodies in blocking solution against α Tubulin (1:250, sc-32293, Santa Cruz) and DNAAF19 (1:100 Thermo Fisher (Invitrogen), PA5-113107) were applied for 2 hour at RT. Secondary antibodies (Supplementary Materials) were applied for 1 hour at RT, before mounting the coverslips onto slides using ProLong Gold Antifade Mountant with DAPI (Invitrogen). Images were taken using a Leica Stellaris 8 confocal.

Pulldown assay

10 µg pEGFP-C1-*hDNAAF19*, pEGFP-C1-*hdnaaf19*^{H154P} or pEGFP-C1 were transfected to HEK293T cells grown on a 10-cm dish in DMEM supplied with 10% FBS and 1x PenStrep using 20 µg of polyethylenimine and incubating the cells overnight. The cells were washed with PBS once, pelleted, then resuspended in a lysis buffer (50 mM Tris-HCl pH7.4, 200 mM NaCl, 5 mM sodium fluoride, 10%(v/v) glycerol, 0.2%(v/v) NP-40, cOmplete, EDTA-free Protease Inhibitor Cocktail (Roche)). The resuspended cells were incubated for 30 min at 4°C on a rotating platform, then cell debris was removed by centrifugation (17,000 g for 10 min at 4°C). 30 µl of in-house made GFP-Trap beads (14) were added to the supernatants, which were incubated for 120 min at 4°C on a rotating platform. The beads were washed with the lysis buffer four times, and bound proteins were released from the beads by denaturing in an SDS-PAGE sample buffer at 98°C. The proteins were separated by SDS-PAGE and transferred to a piece of nitrocellulose membrane for Western blotting, which was stained with ponceau and probed using 1/1000 mouse anti-RUVBL2 (Sigma SAB4200115-25UL, a kind gift from Prof. Colin Johnson (University of Leeds)).

Cryo-EM data collection

The hDNAAF19-RUVBL1/2 complex was diluted to 13.5 µM in 20 mM HEPES (pH 7.5), 250 mM NaCl, and 2 mM DTT. 187 µM of DNAAF19-RUVBL1/2 was mixed with a 3 molar excess of DPCD and left on ice for ~1 hour. The premixed DNAAF19-RUVBL1/2-DPCD was diluted 2-fold with 20 mM Hepes, pH 7.5, 250 mM NaCl, and 2 mM DTT just before being blotted onto cryo-EM grids. Both samples were blotted onto a Quantifoil R1.2/1.3 300 mesh Cu grid, which was glow-discharged using Easiglow (12 mA for 100 sec), using a Vitrobot IV (blot force one and blot time 6 sec at 4 °C with 100% humidity). Micrographs were collected using Titan Krios electron microscopes equipped with Falcon 4i (ThermoFisher Scientific). The data collection parameters are shown in SI Appendix, Table S3).

Data processing

The overall cryo-EM workflow of the hDNAAF19-RUVBL1/2 data is shown in SI Appendix, Fig. S6A. The motion correction of the micrographs was performed using MotionCor2 (19). The contrast transfer function (CTF) of each micrograph was estimated using CTFFIND4 (20). The micrographs were imported into RELION4 (21) and particles were picked using the Auto-picking module and extracted with a box size of 280 x 280 pixels. The extracted particles were imported to CryoSPARC (22) and subjected to 2D classification. The 2D classes with defined structure features were selected and used for ab initio reconstruction of four models. The heterogeneous refinement module was used to refine the particles for the ab initio reconstruction against the four models. The best structure was selected and refined using the non-uniform refinement module. The refined particle set was aligned along the C6 symmetry axis and subjected to symmetry expansion in cryoSPARC. The resulting particles were then separated according to symmetry operators. These six particle sets were converted into STAR files using PyEM (23) and subsequently used for focused 3D classification in RELION5 (24). Focused masks targeting hDNAAF19 and the RUVBL core domains were generated using ChimeraX (25) and RELION5. The 3D classification outcomes are shown in SI Appendix, Fig. S7A, B. Homogeneous reconstructions of two classes, distinguished by the presence or absence of hDNAAF19 density following 3D classification of the RUVBL core (SI Appendix, Fig. S7B), were carried out using CryoSPARC.

As for the hDNAAF19-RUVBL1/2-DPCD data, the overall cryo-EM workflow is shown in SI Appendix, Fig. S6A. The movies were imported to CryoSPARC for Patch Motion Correction. The motion-corrected micrographs were subjected to Patch CTF Estimation. 516 particles were manually picked and subjected to 2D classification. Six structurally defined 2D classes were used for Template Picker across all micrographs, and particles were extracted with a box size of 460 x 460 pixels and subjected to 2D classification. Good 2D classes were selected and used to reconstruct three models by ab initio. The best model was selected and used to generate 50 template 2D classes for Template Picker after non-uniform refinement. The picked particles were a box size of 230 x 230 pixels in bin 2 (1.48 Å/pixel) and were subjected to 2D classification. Good 2D classes were selected, and the *ab initio* reconstruction of three models was performed. Two models and their particles were used for heterogeneous refinement. The best model and its particles were used for homogeneous and non-uniform refinement. After correcting the handedness and aligning the particles along the C6 symmetry axis, the data were subjected to symmetry expansion, and particles corresponding to each C6 symmetry operator were extracted.

Masks were generated around hDNAAF19 and hDPCD together with the external DII domain of hRUVBL2 using ChimeraX and cryoSPARC. These masks were applied for local refinement of each symmetry-

expanded particle set in cryoSPARC. Both the original symmetry-expanded particle sets and the locally refined particles were then converted into STAR files using PyEM and used for focused 3D classification of (i) hDNAAF19, (ii) hDPCD and the external DII domain of hRUVBL2, and (iii) the core domains of RUVBL in RELION5. The 3D classification outcomes are shown in SI Appendix, Fig. S7C, D. The major 3D classes corresponding to hDPCD with the external DII domains of the five hRUVBL2 subunits, which showed clear density, were imported into cryoSPARC for local refinement. In parallel, local refinement was performed on the six RUVBL core domains of the hDNAAF19-RUVBL1/2-DPCD hexamer, in which a single hDNAAF19 molecule interacts with the hRUVBL2 subunit A. The resulting locally refined maps were subsequently combined using PHENIX (26) to generate a composite map. Homogeneous reconstructions of two classes, characterized by the absence of hDNAAF19 density after 3D classification of the RUVBL core (SI Appendix, Fig. S7D), were performed using CryoSPARC. The resolution of each map was determined by the gold standard 0.143 Fourier shell correlation (FSC) criterion using CryoSPARC. The local resolutions of consensus and composite maps were calculated using CryoSPARC. The model details are shown in SI Appendix, Table S4.

Model building

The initial hDNAAF19-RUVBL1/2 model, containing one hRUVBL1, five RhUVBL2, and one DNAAF19, was generated using AlphaFold3 (27). The model was fitted onto the cryo-EM map of DNAAF19-RUVBL1/2 using Chimera X (25). The external DII domains of hRUVBLs were removed on Coot (28). The real-space refinement of the model against the sharpened map from CryoSPARC was carried out using phenix.real_space_refine module (26). The refined model was manually refined against the map using Coot. The B-factors of the final models were refined against the map using Phenix, and the model was validated using Phenix.

The hDNAAF19-RUVBL1/2 model was used as the initial model for hDNAAF19-RUVBL1/2-DPCD and refined in a manner similar to hDNAAF19-RUVBL1/2. The structure of the hDNAAF19-RUVBL1/2 model was fitted onto the focused map of hDNAAF19-RUVBL1/2 core. After a round of phenix.real_space_refine, the model was manually refined in Coot using the map sharpened in CryoSPARC. Separately, an AlphaFold3 model of the external DII domain in complex with DPCD was fitted onto the focused maps of the region for subunits A-E of hRUVBL2 on Chimera X. All the side chains were removed, and the parts that were not observed in the map were deleted in Coot. The external DII/hDPCD models were combined with the refined hDNAAF19-RUVBL1/2 core on the combined map using Coot. Finally, the B-factors of the final model were refined against the combined map using Phenix, and the model was validated using Phenix.

Native mass spectrometry

Purified hDNAAF103 was exchanged into a volatile solution (100 mM ammonium acetate) using a centrifugal exchange device (Micro Bio-Spin 6, Bio-Rad) according to the manufacturer's instructions. Native mass spectrometry experiments were performed on the Q-Exactive Plus UHMR (Thermo Fisher Scientific). Each sample was loaded into homemade Palladium-gold (Pd-Au) coated borosilicate glass capillaries and mounted onto a Q-Exactive Plus UHMR (Thermo) mass spectrometer, where native mass spectrometry experiments were performed. Nano-spray ionisation source (NSI) was used, and generated protein ions were drawn into the vacuum of the mass spectrometer. Instrument parameters were carefully optimised to avoid ion activation and protein unfolding. The UHMR settings used were: 1.5 kV spray voltage, capillary temperature 250 °C, IST off, no extended trapping, micro scan count of 5, Injection time of 100ms. Data were processed and analysed using MassLynx v.4.1 (Waters) and UniDec (v.4.2.2).

Circular dichroism

0.5 mg/ml of purified recombinant hDNAAF19 was dialysed against 100 mM sodium phosphate buffer, pH 7.4, at 4 ºC overnight. After dialysis, the protein concentration was adjusted to 0.3 mg/ml using the phosphate buffer. CD spectra were acquired in the 180-260 nm range on a Chirascan CD Spectrometer (Applied Photophysics) using a 2 nm bandwidth and 1 mm path length cuvettes at 20 ºC. Thermal melting was performed from 20 to 90°C at 1°C/min over the spectral range of 180-260 nm. The spectra were analysed using Pro-Data and Global 3 Analysis software (Applied Photophysics)(29).

SI References

1. E. Krissinel, K. Henrick, Inference of Macromolecular Assemblies from Crystalline State. *J. Mol. Biol.* **372**, 774–797 (2007).
2. R. Pereira, *et al.*, Characterization of CCDC103 expression profiles: further insights in primary ciliary dyskinesia and in human reproduction. *Journal of Assisted Reproduction and Genetics 2019 36:8* **36**, 1683–1700 (2019).
3. Z. Guo, W. Chen, L. Wang, L. Qian, Clinical and Genetic Spectrum of Children with Primary Ciliary Dyskinesia in China. *Journal of Pediatrics* **225**, 157-165.e5 (2020).
4. M. R. Fassad, *et al.*, Clinical utility of NGS diagnosis and disease stratification in a multiethnic primary ciliary dyskinesia cohort. *J. Med. Genet.* **57**, 322–330 (2020).
5. A. Shoemark, *et al.*, High prevalence of CCDC103 p.His154Pro mutation causing primary ciliary dyskinesia disrupts protein oligomerisation and is associated with normal diagnostic investigations. *Thorax* **73**, 157–166 (2018).
6. B. O. Staar, *et al.*, Ciliary Ultrastructure Assessed by Transmission Electron Microscopy in Adults with Bronchiectasis and Suspected Primary Ciliary Dyskinesia but Inconclusive Genotype. *Cells* **12**, 2651 (2023).
7. F. U. Kahraman, *et al.*, Evaluation of the Clinical and Genetic Characteristics of Primary Ciliary Dyskinesia Patients With Situs Inversus Totalis. *Birth Defects Res.* **117**, e2444 (2025).
8. R. Pereira, *et al.*, Characterization of CCDC103 expression profiles: further insights in primary ciliary dyskinesia and in human reproduction. *Journal of Assisted Reproduction and Genetics 2019 36:8* **36**, 1683–1700 (2019).
9. W. A. Houry, E. Bertrand, B. Coulombe, The PAQosome, an R2TP-Based Chaperone for Quaternary Structure Formation. *Trends Biochem. Sci.* **43**, 4–9 (2018).
10. J. Lynham, W. A. Houry, The Role of Hsp90-R2TP in Macromolecular Complex Assembly and Stabilization. *Biomolecules 2022, Vol. 12, Page 1045* **12**, 1045 (2022).
11. J. Lynham, W. A. Houry, The Multiple Functions of the PAQosome: An R2TP- and URI1 Prefoldin-Based Chaperone Complex. *Adv. Exp. Med. Biol.* **1106**, 37–72 (2018).
12. M. A. Zariwala, K. A. Despotes, S. D. Davis, Primary Ciliary Dyskinesia. *GeneReviews®* (2025).
13. B. Braschi, *et al.*, Consensus nomenclature for dyneins and associated assembly factors. *Journal of Cell Biology* **221** (2022).
14. T. Ochi, *et al.*, CCDC61/VFL3 Is a Paralog of SAS6 and Promotes Ciliary Functions. *Structure* **28**, 674–689 (2020).
15. J. Peränen, M. Rikkonen, M. Hyvönen, L. Kääriäinen, T7 Vectors with a Modified T7lacPromoter for Expression of Proteins inEscherichia coli. *Anal. Biochem.* **236**, 371–373 (1996).
16. D. J. Merk, *et al.*, Functional screening reveals genetic dependencies and diverging cell cycle control in atypical teratoid rhabdoid tumors. *Genome Biology 2024 25:1* **25**, 301- (2024).
17. V. Demichev, C. B. Messner, S. I. Vernardis, K. S. Lilley, M. Ralser, DIA-NN: neural networks and interference correction enable deep proteome coverage in high throughput. *Nature Methods 2019 17:1* **17**, 41–44 (2019).
18. R. Maeshima, A. I. Jacobs, M. T. Dalbay, S. L. Hart, BMI1 Transduction of Human Airway Epithelial Cells for Expansion of Proliferation and Differentiation. *Methods in Molecular Biology* **2725**, 225–237 (2024).
19. S. Q. Zheng, *et al.*, MotionCor2: anisotropic correction of beam-induced motion for improved cryo-electron microscopy. *Nature Methods 2017 14:4* **14**, 331–332 (2017).
20. A. Rohou, N. Grigorieff, CTFFIND4: Fast and accurate defocus estimation from electron micrographs. *J. Struct. Biol.* **192**, 216–221 (2015).
21. D. Kimanius, L. Dong, G. Sharov, T. Nakane, S. H. W. Scheres, New tools for automated cryo-EM single-particle analysis in RELION-4.0. *Biochemical Journal* **478**, 4169–4185 (2021).
22. A. Punjani, J. L. Rubinstein, D. J. Fleet, M. A. Brubaker, cryoSPARC: algorithms for rapid unsupervised cryo-EM structure determination. *Nature Methods 2017 14:3* **14**, 290–296 (2017).
23. D. Asarnow, E. Palovcak, Y. Cheng, asarnow/pyem: UCSF pyem v0.5. https://doi.org/10.5281/ZENODO.3576630.
24. A. Burt, *et al.*, An image processing pipeline for electron cryo-tomography in RELION-5. *FEBS Open Bio* **14**, 1788–1804 (2024).
25. E. C. Meng, *et al.*, UCSF ChimeraX: Tools for structure building and analysis. *Protein Science* **32**, e4792 (2023).
26. D. Liebschner, *et al.*, Macromolecular structure determination using X-rays, neutrons and electrons: recent developments in Phenix. *urn:issn:2059-7983* **75**, 861–877 (2019).
27. J. Abramson, *et al.*, Accurate structure prediction of biomolecular interactions with AlphaFold 3. *Nature 2024 630:8016* **630**, 493–500 (2024).

28. P. Emsley, B. Lohkamp, W. Scott, K. Cowtan, Features and development of Coot. *Acta Crystallogr. D Biol. Crystallogr.* **66**, 486–501 (2010).
29. M. T. Marty, *et al.*, Bayesian deconvolution of mass and ion mobility spectra: From binary interactions to polydisperse ensembles. *Anal. Chem.* **87**, 4370–4376 (2015).
